# Investigating the Role of Metabolism for Antibiotic Combination Therapies in *Pseudomonas aeruginosa*

**DOI:** 10.1021/acsinfecdis.3c00452

**Published:** 2023-11-08

**Authors:** Martina M. Golden, Savannah J. Post, Renata Rivera, William M. Wuest

**Affiliations:** † Department of Chemistry, Emory University, Atlanta, Georgia 30322, United States; ‡ Emory Antibiotic Resistance Center, Emory School of Medicine, Emory University, Atlanta, Georgia 30322, United States

**Keywords:** combination studies, metabolism, synergy, antibiotics, pseudomonas, resistance

## Abstract

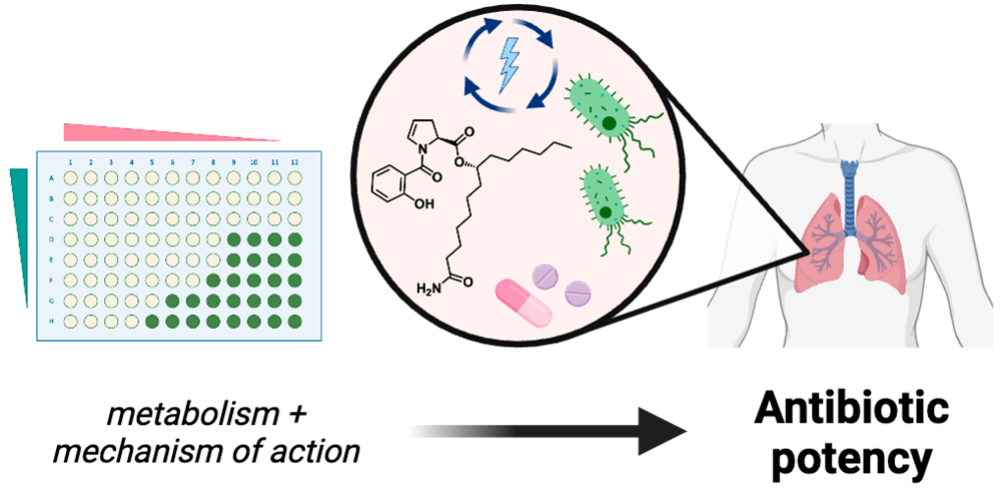

Antibacterial resistance poses a severe threat to public health; an anticipated 14-fold increase in multidrug-resistant (MDR) bacterial infections is expected to occur by 2050. Contrary to antibiotics, combination therapies are the standard of care for antiviral and anticancer treatments, as synergistic drug–drug interactions can decrease dosage and resistance development. In this study, we investigated combination treatments of a novel succinate dehydrogenase inhibitor (promysalin) with specific inhibitors of metabolism and efflux alongside a panel of clinically approved antibiotics in synergy studies. Through these investigations, we determined that promysalin can work synergistically with vancomycin and antagonistically with aminoglycosides and a glyoxylate shunt pathway inhibitor at subinhibitory concentrations; however, these cooperative effects do not reduce minimum inhibitory concentrations. The variability of these results underscores the complexity of targeting metabolism for combination therapies in antibiotic development.

In the early 1900s, bacterial infections frequently led to grave outcomes, such as amputation and death. However, the rise of antibiotics revolutionized healthcare, beginning with the use of sulfonamides, the first broad-spectrum agent that was used clinically.^1^ Shortly thereafter, the initial discovery of penicillin in 1928 sparked the “Golden Age” of antibiotic discovery.^2^ Between 1940 and 1970, nearly 30 new classes of antibiotics were discovered, comprising great structural diversity and spanning five distinct mechanisms of action. Many of these antibiotics have made a significant impact in the healthcare^3^ and agriculture^4^ industries. However, their over- and misuse have accelerated the spread of bacterial resistance^5−7^ and have therefore reduced their effectiveness. Furthermore, only five new classes of antibiotics have been introduced to the clinic since 2000, with only one functioning through a novel mechanism of action.^1^ Together, these factors have contributed to a rapid rise in antibiotic-resistant infections. According to the Centers for Disease Control and Prevention (CDC), 2.8 million people in the United States alone contract antibiotic-resistant infections each year, resulting in 35 000 deaths.^9^ One particularly threatening pathogen is *Pseudomonas aeruginosa*, which is responsible for 2700 antibiotic resistance-associated deaths every year and is the most common causative bacteria in cystic fibrosis-related chronic infections.^9^

To combat the issue of antibiotic resistance, our group has focused on two strategies: narrow-spectrum small molecules and synergistic combinations. Narrow-spectrum compounds are advantageous because they allow for the selective killing of the pathogen; therefore, they do not inhibit the growth of commensal bacteria.^10^ Combination therapies offer potentially beneficial drug–drug interactions, such as the clinical combination of sulfamethoxazole (SMX) and trimethoprim (TMP), which exhibits synergism. This type of strategy has shown great success in cancer treatment^11^ and antivirals,^12^ among others, and has become the standard treatment regime for such diseases.^13^ However, combinations in antibacterials are less common despite their significant therapeutic potential, owing to the difficulty of identifying synergistic combinations of therapies. Accordingly, identifying new combination therapies that leverage novel mechanisms of action would drastically reduce the likelihood of clinical resistance.

In this study, we selected the natural product promysalin (**1**), which has been the subject of much prior work in our group, as a potential candidate for a narrow-spectrum antibacterial component in combination therapy. This natural product was first isolated by De Mot and co-workers, who identified its uniquely selective activity against *Pseudomonas aeruginosa*.^14^ Intrigued by this activity, our lab completed the first total synthesis^15^ and several panels of analogues, where we explored the structure–activity relationships (SARs) for nearly all regions of the molecule.^16−18^ Furthermore, our previous work has shown that, despite having an excellent (half-maximal inhibitory concentration) IC_50_ (3 nM or 1.4 ng/mL for the PA14 strain) against *P. aeruginosa*, promysalin has a remarkably high minimal inhibitory concentration (MIC) of >250 μM (Figure S1). The difference between the two values lies in the percent of inhibited bacterial growth; IC_50_ represents 50% growth inhibition, while MIC is the lowest concentration where there is no visible bacteria growth. Following an initial screening, our lab identified its biological target as succinate dehydrogenase (Sdh) in *P. aeruginosa* without a significant activity toward mammalian Sdh.^19^ Importantly, this is a distinct molecular target from current clinical antibiotics, and we speculated that this compound could be used as a chemical probe to motivate these studies.

Our initial efforts sought to narrow the gap between the IC_50_ and MIC of promysalin. We hypothesized that this gap was caused by an innate resistance mechanism(s) in *P. aeruginosa*, which prevented the full inhibition of growth. To investigate this, we tested the inhibitors of several resistance pathways for synergistic inhibition of *P. aeruginosa* growth. This campaign began with the synthesis of promysalin, which was accomplished in a highly convergent manner that was amenable to analogue synthesis. Previous works have shown that a simplified promysalin analogue, **2**, was similar in activity, had the same mechanism of action,^19^ and was synthetically more accessible,^16^ as it only differed from the natural product in the absence of the alcohol side chain (Figure 1), which requires an additional four reactions to install. Therefore, we completed the previously reported simplified synthesis of **2** (Scheme S1) and confirmed its inhibitory activity (Figure S1), setting the stage for the experiments described herein.

**Figure 1:**
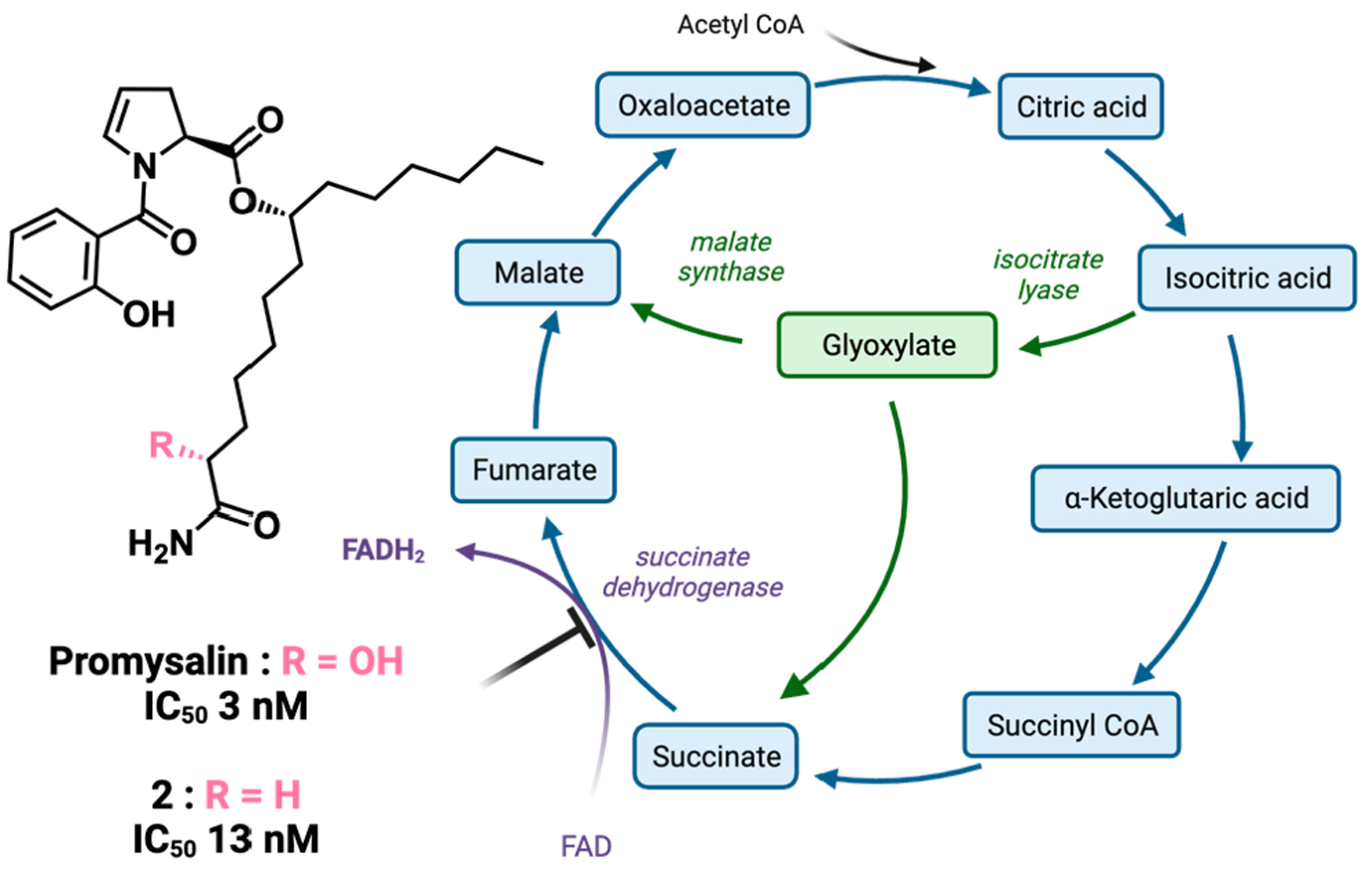
IC_50_ values of promysalin and **2** and the inhibition of succinate dehydrogenase in the TCA cycle (blue) and the glyoxylate shunt pathway (green). The 3 nM concentration of promysalin was 1.4 ng/mL, and the 13 nM concentration of **2** was 6 ng/mL.

Our initial efforts sought to inhibit possible resistance mechanisms,^20^ as we hypothesized that an innate resistance was responsible for the stark difference between the MIC and IC_50_ values of promysalin and aimed to identify and mitigate the resistance pathway(s). We first considered efflux as an innate resistance mechanism through which *P. aeruginosa* may be evading promysalin treatment. To explore this hypothesis, we first sought to employ efflux pump inhibitors (EPIs) in combination with **2**. Because this strategy would not allow us to calculate standard fractional inhibitory concentration (FIC) indexes, as the inhibitors did not reduce *P. aeruginosa* growth at the tested concentrations, we analyzed the changes in the IC_50_ of **2** to determine the interaction of the compounds. To this end, we considered the EPIs carbonyl cyanide *m*-chlorophenyl hydrazone (CCCP) and phenylalanine-arginine β-naphthylamide (PAβN) (Figure 2A). CCCP acts as a protonophore and is known to globally decrease efflux pump effectiveness through the disruption of the proton gradient, which is required for the function of most efflux pumps.^21^ However, we saw that it had no effect on the activity of **2**, when tested in combination, up to the solubility limit. As we considered the mechanism of CCCP, we thought that, while the disruption of the proton gradient diminished the efflux, it could also diminish the active transport of **2** into the cells, thus negating any potential effects of the efflux inhibition. PAβN is known to be an outer membrane permeabilizer and an EPI in Gram-negative bacteria.^22^ At higher concentrations of PAβN in combination with **2**, we observed a decrease in the IC_50_ of **2** in a PAβN dose-dependent manner, as well as an overall decrease in growth (Figure 2B,C). Concurrently in our laboratory, it was discovered that **2** shows a 10 000-fold decrease in IC_50_ (516 nM to 74 pM) in a *P. aeruginosa* efflux knock out strain, where eight efflux pumps and porins are absent, which have been shown to contribute to antibiotic resistance in *P. aeruginosa*.^23^ However, complete growth inhibition was again not observed,^24^ suggesting that there are mechanisms other than efflux by which *P. aeruginosa* evades treatment with **2**, most likely seemingly through metabolic perturbation.

**Figure 2:**
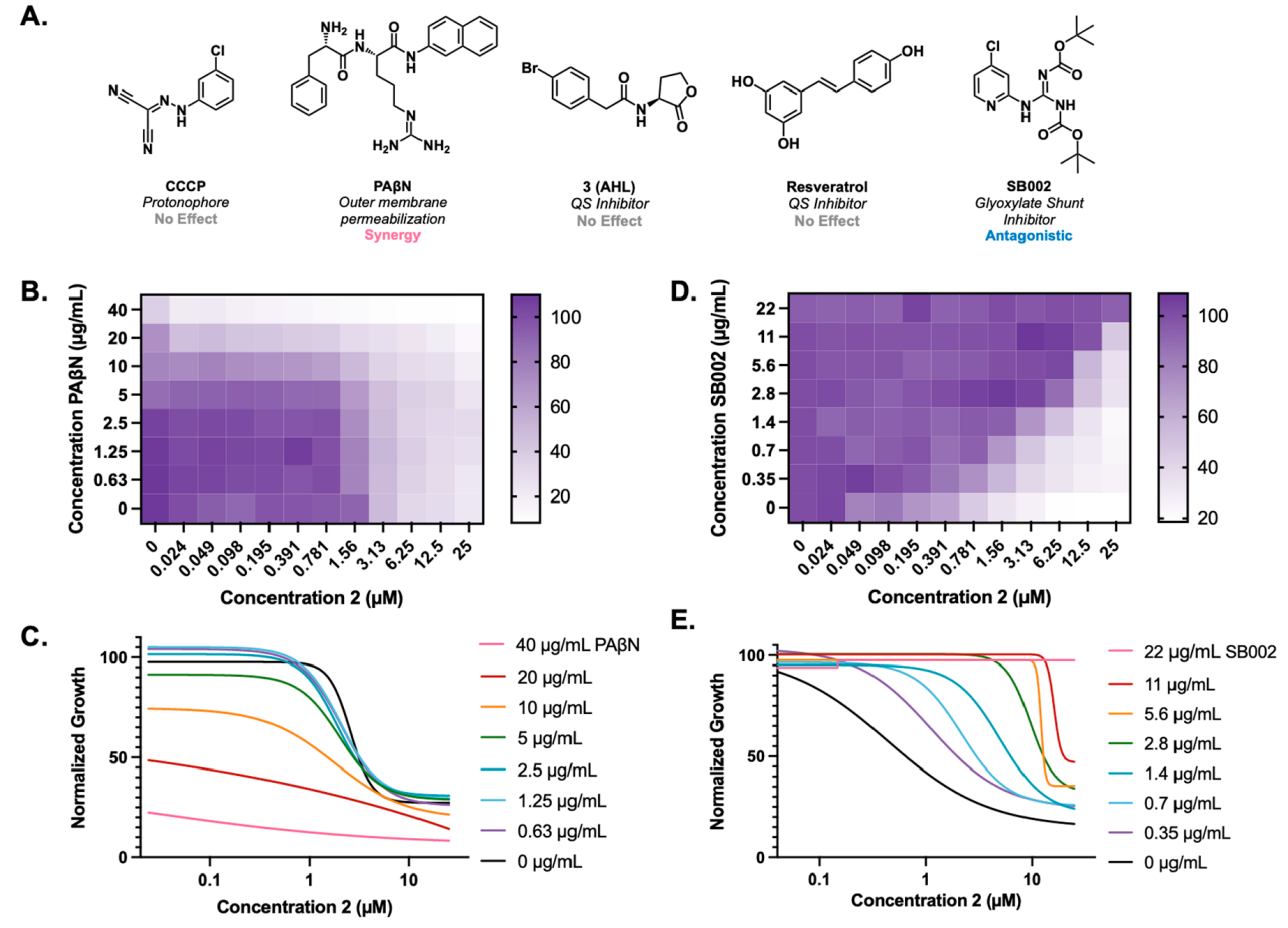
Growth inhibition of *P. aeruginosa* PA14 with different inhibitors. (A) Structures of the compounds tested in combination with **2**, with their respective mechanisms of action and the effect of the treatment on the activity. (B) Combination growth inhibition with varying concentrations of **2** and PAβN. (C) Growth inhibition curves of the combination of PAβN and **2**. (D) Combination growth inhibition with varying concentrations of **2** and SB002. (E) Growth inhibition curves of the combination of SB002 and **2**.

In addition to innate resistance, *P. aeruginosa* can also exhibit adaptive resistance, wherein it makes transient changes to the expression of specific genes or proteins in response to an external stimulus or threat. One example is quorum sensing, where bacteria use small molecules to sense population density and signal how to respond to threats, such as transitioning to a persistent state or producing biofilm.^25,26^ Thus, we questioned if adaptive resistance via quorum sensing (QS) hindered promysalin activity and sought to examine the effects of combining **2** with known QS inhibitors. We first considered *N*-acyl-L-homoserine lactones (AHLs), which are small molecules that are used by many Gram-negative bacteria, including *P. aeruginosa,* for various signaling purposes. We selected a derivative identified by the Blackwell lab, **3** (Figure 2A), as it has been shown to be a more potent AHL analogue.^27^ However, when tested in a checkerboard assay, it had no effect on the IC_50_ of **2**, indicating that this quorum sensing pathway likely does not impede the activity of promysalin. We then considered alternative QS pathways, as these signaling pathways are important for pathogenicity. Resveratrol (Figure 2A) was selected to investigate this possibility, as it has previously shown inhibition of several different QS pathways in *P. aeruginosa*.^28^ Again, no effect was observed, suggesting that these common signaling pathways are not responsible for the promysalin resistance of *P. aeruginosa*.

Lastly, when thinking about promysalin’s mechanism more specifically in regard to how it targets Sdh, an enzyme in the tricarboxylic acid cycle (TCA cycle), we looked to the literature for other examples of metabolism inhibitors. One of the key challenges in targeting bacterial metabolism is the complexity and interconnectivity of the various metabolic pathways. The glyoxylate shunt pathway specifically bypasses Sdh (Figure 1) in the TCA cycle.^29^ Our earlier work hinted at *P. aeruginosa* diverting metabolic flux through this pathway to overcome promysalin treatment, as this was also found to be the case in *Pseudomonas putida*.^30^ Notably, Sdh is also utilized in the electron transport chain and is critical for the production of adenosine triphosphate (ATP); therefore, we presumed that diverting to the shunt pathway would come at a high fitness cost, which could explain the low levels of growth relative to the negative controls. We postulated that if we could inhibit the canonical TCA cycle and the shunt pathway simultaneously, we could fully inhibit the primary metabolism and achieve full growth inhibition at lower concentrations. Thus, we searched for reported glyoxylate shunt inhibitors to test in combination with promysalin. Itaconate was initially considered, as it is produced by human cells and inhibits isocitrate lyase, the first enzyme in the glyoxylate shunt.^31^ However, it was not selected because *P. aeruginosa* can degrade this molecule.^32^ SB002 was then identified, which has exhibited the potent inhibition of malate synthase and isocitrate lyase, which are both enzymes comprising the glyoxylate shunt pathway (Figure 1).^33^ Subsequently, SB002 was synthesized in one step according to the reported procedure (see the Supporting Information) and was tested in a checkerboard assay with **2** (Figure 2D). To our surprise, SB002 diminished the inhibitory activity of **2** in a concentration-dependent manner (Figure 2D). We postulated that the presence of SB002 could trigger the overall upregulation of metabolism, resulting in the increased expression of Sdh and thus mitigating the effect of **2**. Further investigations exploring this hypothesis are currently underway, and at this point, we pivoted our focus toward testing **2** with clinically approved antibiotics.

As previously discussed, novel mechanisms of action are crucial to preventing antibiotic resistance. Therefore, we aimed to combine **2** with clinically approved antibiotics, as it has a novel mechanism of action that could prove beneficial for long-term resistance prevention. A panel of 15 traditional antibiotics spanning all major mechanisms of action was employed (Table 1). Each antibiotic was tested in combination with **2** against *P. aeruginosa* PA14 in a standard serial dilution checkerboard assay. Typically, visual MICs are used to analyze these results, indicating either synergistic, antagonistic, or no compound interactions.^34^ However, although promysalin exhibits a very potent IC_50_ against *P. aeruginosa*, the MIC is much higher. Thus, we instead measured the optical density and calculated the IC_50_ values for each combination. These values were then used to calculate an FIC_50_ index for each combination (eq S1) in place of the traditional FIC index. The calculated FIC_50_ values were then used to inform future checkerboard MIC assays to validate the growth patterns observed at 50% growth inhibition.

**Table 1:** Results of Combinations of Traditional Antibiotics with **2**, Including Their Antibiotic Class, Mechanism of Action, Category, FIC_50_ Index, and Interaction

antibiotic	class	mechanism	category	FIC_50_ index^a^_,_^b^	interaction^c^
vancomycin	glycopeptide	cell wall synthesis	bacteriostatic	0.3–0.4	synergism
chloramphenicol	-	protein synthesis (50S)	bacteriostatic	0.6–1.7	no interaction
tetracycline	tetracycline	protein synthesis (30S)	bacteriostatic	1.2–2.2	no interaction
erythromycin	macrolide	protein synthesis (50S)	bacteriostatic	1.3–2.4	no interaction
trimethoprim	-	folate synthesis	bacteriostatic	1.4–2.5	no interaction
piperacillin	penicillin	cell wall synthesis	bactericidal	2.4–3.4	no interaction
ciprofloxacin	fluoroquinolone	DNA synthesis	bactericidal	2.3–3.9	no interaction
levofloxacin	fluoroquinolone	DNA synthesis	bactericidal	2.2–3.9	no interaction
colistin	polymyxin	cell membrane	bactericidal	1.3–3.9	no interaction
polymyxin B	polymyxin	cell membrane	bactericidal	3.5–5.6	antagonism
ceftazidime	cephalosporin	cell wall synthesis	bactericidal	5.4–11	antagonism
tobramycin	aminoglycoside	protein synthesis (30S)	bactericidal	5.3–13	antagonism
gentamicin	aminoglycoside	protein synthesis (30S)	bactericidal	3.7–6.3	antagonism
amikacin	aminoglycoside	protein synthesis (30S)	bactericidal	4.3–10	antagonism
kanamycin	aminoglycoside	protein synthesis (30S)	bactericidal	5.4–21	antagonism

a Each checkerboard assay was completed in biological triplicate.

b The FIC_50_ range represent the range of high and low values from the replicates.

c Standard metrics for drug interactions were utilized, where FIC_50_ < 0.5 indicates synergy, 0.5 ≤ FIC_50_ ≤ 4 indicates additivity, and FIC_50_ > 4 indicates antagonism.

Upon the completion of the checkerboard assays and the FIC_50_ calculations for **2** with the 15 antibiotics, we found a synergistic relationship between **2** and vancomycin (0.3 ≤ FIC_50_ ≤ 0.4), a primarily Gram-positive antibiotic (Table 1 and Figure 3A). To determine if **2** was permeabilizing the outer membrane, thus sensitizing *P. aeruginosa* to vancomycin, we performed permeabilization assays with the fluorescent probe *N*-phenylnaphthalen-1-amine (NPN) with vancomycin, **2**, and combinations of the two. NPN does not fluoresce significantly in water, but when the outer membrane is permeabilized, it can access the cytosolic membrane and fluoresce in the hydrophobic environment.^35^ This revealed that **2** does not permeabilize the outer membrane alone and that the combination of the two compounds does not differ from vancomycin alone (Figure 3B). Recent literature has shown that *P. aeruginosa* can be sensitized to vancomycin through nutrient limitation and that these effects are dependent on the concentration of Fe^3+^ and Cu^2+^.^36^ The inhibition of Sdh affects both carbon metabolism and the electron transport chain, which is important for iron homeostasis. Previous work in our laboratory focused on the transcriptomic changes of *P. putida* during promysalin treatment, and the downregulation of bacterioferritin genes was noted, which suggests that the cells experienced iron-limiting conditions despite being in an iron-rich medium (TSB).^30^ With the treatment of **2**, *P. aeruginosa* could be experiencing the same iron-related transcriptional changes that could sensitize the bacteria to vancomycin treatment. Future studies will aim to determine the exact nature of this synergistic relationship.

**Figure 3:**
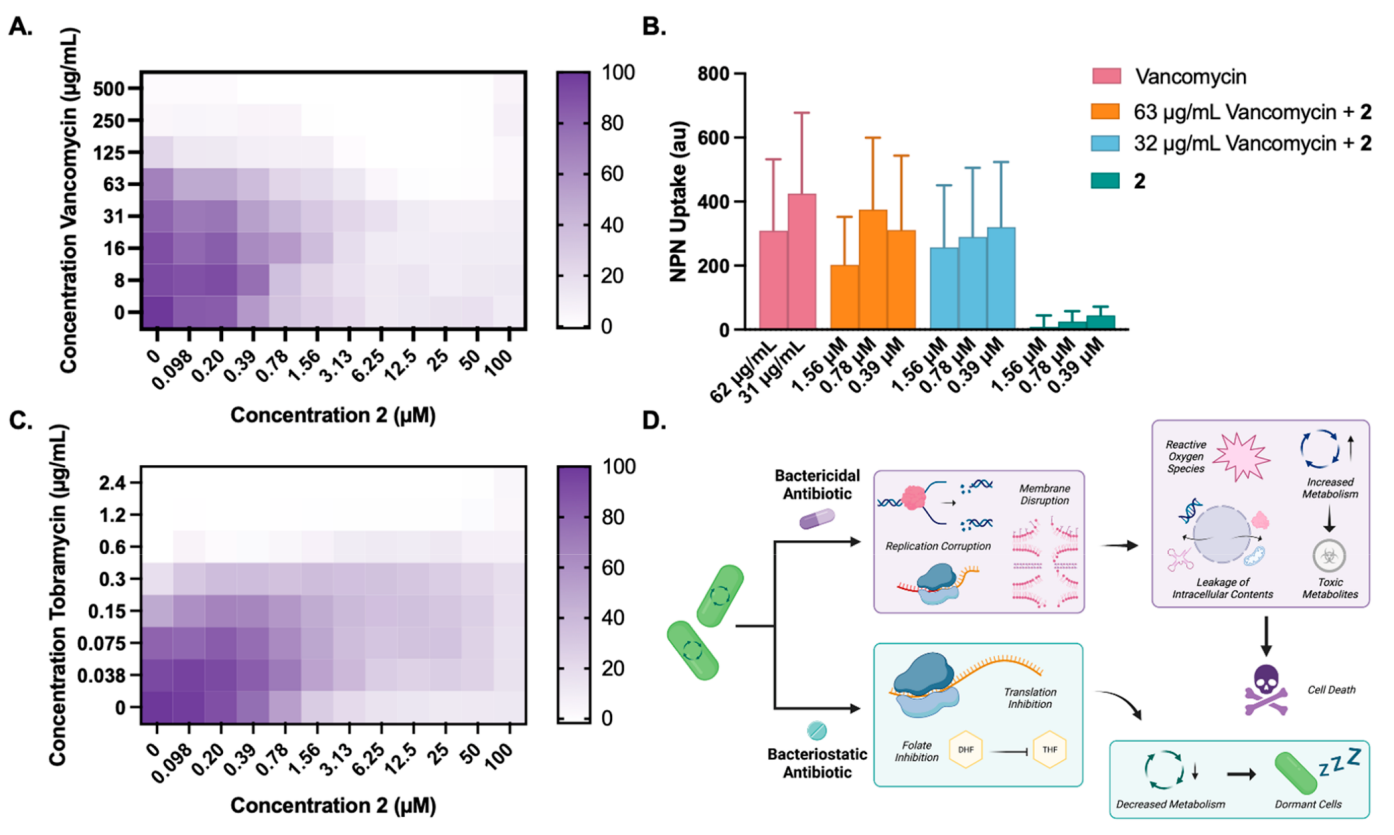
Summary of the combination study results with **2** and the antibiotic panel. (A) Combination growth inhibition of **2** and vancomycin. (B) NPN uptake assay with vancomycin, **2**, and combinations of the two compounds: **2** concentrations of 1.56, 0.78, and 0.39 μM convert to 0.72, 0.36, and 0.18 μg/mL, respectively. (C) Combination growth inhibition of **2** and tobramycin. (D) Differences in the mechanisms of the bactericidal and bacteriostatic antibiotics and their effects on cells.

With the hope of decreasing the toxicity associated with polymyxin antibiotics,^37^ we tested colistin and polymyxin B in combination with **2**. However, to our surprise, we did not see a significant interaction between the compounds. *P. aeruginosa* respiratory infections are commonly treated with aminoglycoside antibiotics; therefore, we tested four aminoglycosides in combination with **2**. Interestingly, an antagonistic interaction was observed for the combination of **2** with each aminoglycoside in the panel (Figure 3C). Additionally, an antagonistic relationship was observed with ceftazidime, a cephalosporin antibiotic that is commonly used to treat Gram-negative infections. Because piperacillin, another β-lactam, showed no interaction with **2**, this could suggest that the bactericidal activity of the two has different reliance on cell metabolism. All of the other antibiotic combinations had no interaction with **2**.

Informed by the FIC_50_ values, we performed checkerboard MIC assays to analyze the change in antibiotic MIC when the mixture was in combination with **2**. Because we observed a synergistic relationship between vancomycin and **2** at 50% growth, we hypothesized that this would result in a decrease in the MIC of vancomycin. However, we did not observe a significant change in the MIC for vancomycin. The same type of experimentation and analysis were performed with tobramycin to investigate potential increases in MIC from an antagonistic relationship. Accordingly, we found that the MIC of tobramycin increased from 19 to 78 μg/mL when in combination with 5 μM **2** (2.3 μg/mL). These results show that the metabolism of *P. aeruginosa* greatly affects the response to antibiotics.

Aminoglycosides require active transport into the cytoplasm to execute their bactericidal mechanism of action;^38^ however, this would be inhibited by **2**. Furthermore, antibiotics with bacteriostatic mechanisms of action cause decreased cellular respiration and metabolism,^39^ which describes the mechanism of **2**. When bacteriostatic compounds are used in combination with bactericidal antibiotics, such as aminoglycosides, the bacteriostatic agent tends to dominate.^39,40^ Therefore, downstream processes that result in cell death (e.g., reactive oxygen species, cell damage, and toxic metabolites) are not initiated (Figure 3D).^41^ This essentially negates the killing power of the bactericidal antibiotic, resulting in an augmented effect. These results illustrate the complexity of targeting metabolism and the resilience of *P. aeruginosa*, which warrants further work to understand this complex pathogen.

On the basis of our growth inhibition assays, we hypothesize two reasons for the high MIC of promysalin and the lack of synergy in our MIC assays. One reason is that the *P. aeruginosa* population has become tolerant to antibacterial treatment. Tolerance is described as a transient ability to withstand antibiotic treatment; however, a tolerant population still eventually succumbs to the antibiotic. Another reason is that *P. aeruginosa* transitions to a persistent state. Persistence describes metabolically dormant nondividing cells, which have been linked to the relapse of antibiotic-resistant infection.^42,43^ Compared with conventionally defined resistance, tolerance and persistence are both transient states that can be altered under certain conditions, such as metabolic flux. Because promysalin targets an enzyme in primary metabolism, we saw these as a likely survival mechanism for *P. aeruginosa.* Additionally, this could also explain why many traditional antibiotics had no interaction with **2**, as most of them require active transport to gain entrance to the cell and exert their mechanism of action, which would be diminished in tolerant/persistent cells. Both hypotheses support the idea that *P. aeruginosa* alters its metabolism for survival. Furthermore, other reports have indicated a link between TCA perturbation and a tolerant cell state, giving further credence to this hypothesis.^44^ Taken together, the induction of tolerant/persistent cells through promysalin treatment would explain many of our observations, and experiments to investigate this hypothesis are ongoing.

In conclusion, our results point to a shift in *P. aeruginosa* metabolism that occurs in response to treatment with **2**. First, we addressed potential resistance mechanisms and found that both efflux and metabolic flux likely contribute to the incomplete inhibition of *P. aeruginosa* growth. Second, we investigated antibiotic combinations, where we determined that a metabolic change could aid in inhibition, as seen with the synergistic relationship with vancomycin, or void antibacterial activity, as seen with the antagonistic combination of **2** and ceftazidime or aminoglycosides. Even though full synergistic growth inhibition was not obtained, these results are vital for informing *P. aeruginosa* treatment, as aminoglycosides are a common treatment for patients with cystic fibrosis.^45^ We hypothesize that the observed incomplete growth inhibition upon treatment of **2** could indicate that *P. aeruginosa* shifts into a metabolically dormant state. Previous studies in the group have focused on the transcriptomic changes of *P. putida* treatment with promysalin, which displayed similar shifts in metabolism, thus partially explaining the species-selectivity of the natural product.^30^ Overall, these results support previous findings, indicating that targeting metabolism is complex and results in a multifaceted response from the bacteria, which makes it difficult to target and predict downstream effects. Our work underscores the importance of considering the metabolic state of bacteria during antibiotic treatment, and **2** can serve as a tool compound to chemically induce metabolic changes in future studies. Overall, this work highlights the importance of understanding metabolism in pathogenic species in order to facilitate the discovery of novel synergistic combinations to address the current antibiotic resistance crisis.

## Methods

### Bacteria Strains and Culture Conditions

*P. aeruginosa* (PA14) was acquired from Prof. Joanna B. Goldberg (School of Medicine, Emory University). All strains were streaked onto Tryptic soy agar (TSA) from a freezer stock and incubated overnight (16–24 h). Then, single colonies were picked and grown overnight while being shaken at 37 °C at 200 rpm in Trypticase soy broth (TSB) medium (5 mL). The overnight cultures were diluted 1:100 in 5 mL of fresh medium and grown at 37 °C and 200 rpm to an OD_600_ (optical density at 600 nm) reading of ∼0.32 (mid-exponential growth) (measured on a Molecular Devices SpectraMax iD3 plate reader or a BioTek Synergy H1 hybrid plate reader). The bacteria were then diluted to a concentration of 0.004 according to the following equation: (*x* μL regrow culture) × (OD reading) = (0.004) × (volume of diluted bacteria culture needed).

### IC_50_ Assay

The compounds were either dissolved in water, 10% DMSO in water, or 5% Tween 80 5% DMSO in water; the vehicle and concentrations that were used are indicated on the respective data. Then, the compounds were serially diluted 2-fold in water to produce 24 concentrations, yielding 100 μL per well. Negative controls of each compound’s respective vehicle were employed. Next, 100 μL of the diluted bacteria was plated into each well, and the plates were incubated statically for 24 h at 37 °C. OD_600_ readings were taken at this point. The data were normalized to the media control and negative control, and then IC_50_ and IC_90_ values were calculated by fitting the OD_600_ readings versus concentrations with a four-parameter logistic S52 model. The compounds were tested in triplicate from three separate overnight cultures, and the results were averaged.

### Checkerboard Assay

All of the compounds besides SB002 were dissolved in water or 10% DMSO in water; the vehicle and concentrations that were used are indicated on the respective data. SB002 was dissolved in 5% MeOH, 1% Tween 20, and TSB. One compound was serially diluted 2-fold in the vehicle 11 times so that there was no compound in the final column (11 concentrations). The second compound was added to all wells in the first seven rows in decreasing concentrations. Water was added to bring the final volume to 100 μL. Then, 100 μL of the diluted bacteria was plated into each well, and the plates were incubated statically for 24 h at 37 °C. OD_600_ readings were taken at this point, and the growth was normalized to the media control and negative control. IC_50_ values were calculated by fitting the normalized growth verus concentrations with a four-parameter logistic S52 model. Equation S1 was used to determine the compound relationship: synergism (FIC_50_ < 0.5), antagonism (FIC_50_ > 4), or no interaction (0.5 ≤ FIC_50_ ≤ 4). The assay was tested in triplicate from three separate overnight cultures.

### NPN Assay

Overnight cultures of PA14 were diluted 1:100 in fresh medium and allowed to grow to the mid-exponential phase (OD_600_ of 0.5) at 37 °C while being shaken at 200 rpm. The cells were harvested by centrifugation (3900 rpm, 25 °C, 10 min) and washed twice with GHEPES buffer (5 mM HEPES and 5 mM glucose). The cells were resuspended in the assay buffer to a final OD_600_ of 1. Then, 90 μL of the cells were combined with 90 μL of GHEPES with 20 μM NPN (from a 5 mM stock solution of NPN in acetone) in a 96-well optical-bottom black plate and incubated for 20 min. The compounds were diluted, and a 10% DMSO concentration was maintained for all compounds. After 20 min of incubation, 20 μL of the compound or vehicle was added to every well, and the fluorescence was immediately monitored at an exciting wavelength of 350 nm and an emission wavelength of 420 nm for 1 h. Maximum permeabilization (NPN uptake) was determined to be the maximum fluorescence intensity, which was normalized to the background of every well.

## Supporting Information Available

Supporting figures, details of the synthetic procedures, and raw checkerboard data (PDF)
