# Supplementary material for: Investigating the Role of Metabolism for Antibiotic Combination Therapies in Pseudomonas aeruginosa
Source: ACS Infect Dis. 2023 Nov 8;9(12):2386–93. doi: 10.1021/acsinfecdis.3c00452 (PMC10714402; doi:10.1021/acsinfecdis.3c00452)
Supplement: Supplementary file 1 — id3c00452_si_001.pdf [file id3c00452_si_001.pdf]

Supporting Information

Investigating the role of metabolism for antibiotic combination therapies in *Pseudomonas aeruginosa*

Martina M. Golden[1], Savannah J. Post[1], Renata Rivera[1], William M. Wuest[1,2*]

[1]Department of Chemistry, Emory University, Atlanta, GA 30322, USA
[2]Emory Antibiotic Resistance Center, Emory School of Medicine, Emory University, Atlanta GA 30322, USA

*To whom correspondence should be addressed. e-mail: wwuest@emory.edu

Table of Contents

1. **Supporting Figures**
   1.1. **Synthetic Scheme for 2** **S2**
   1.2. **Equation for calculating FIC_{50} index** **S3**
   1.3. **IC_{50} data for promysalin and 2** **S3**

2. **Chemistry**
   2.1. **General Notes** **S3**
   2.2. **Procedures and Characterization** **S4-5**

3. **Biology**
   3.1. **Raw Antibiotic Combination Data** **S6-14**

4. **References** **S14**

1. Supporting Figures

1.1. Synthetic Scheme for 2

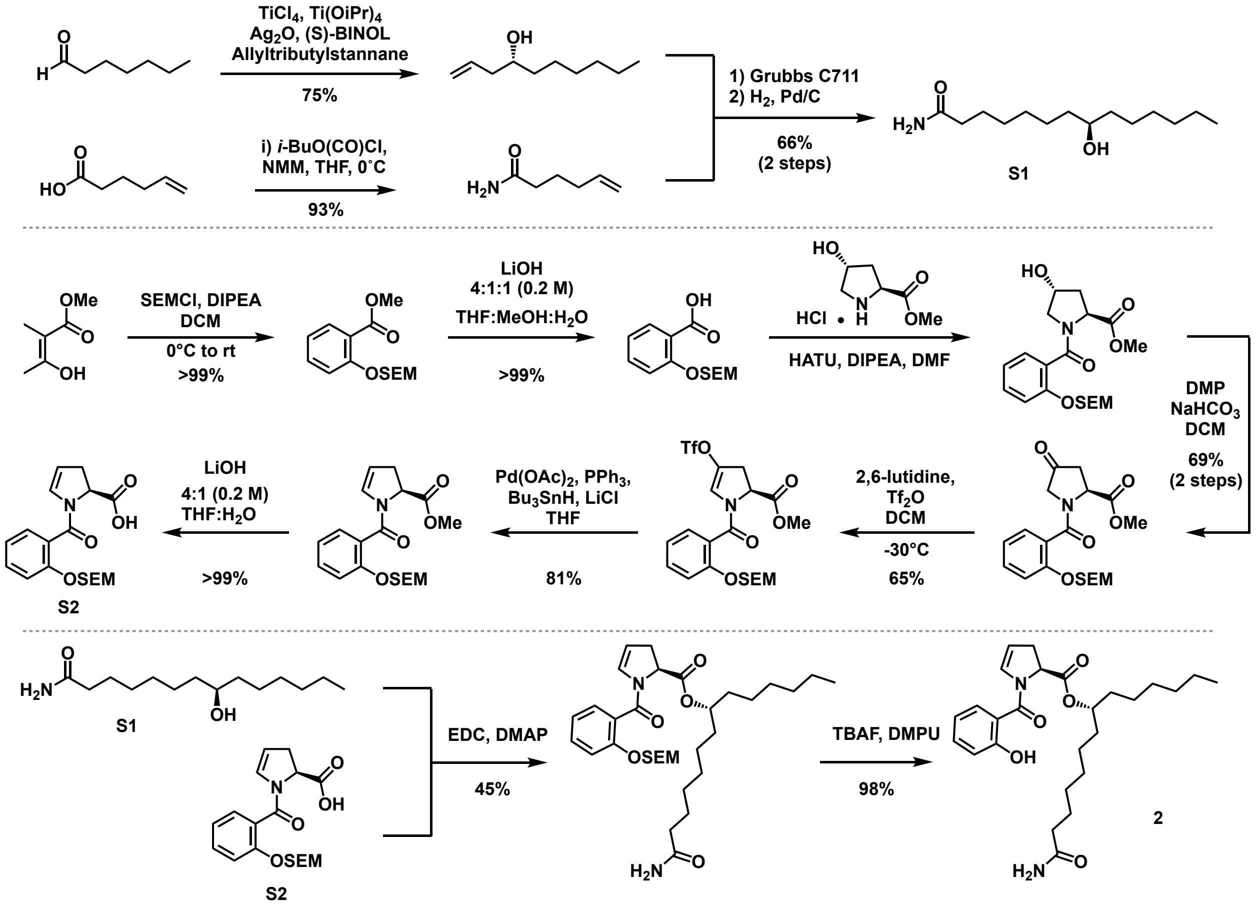

Scheme S1. Synthesis of simplified promysalin analog, **2**. Full procedures and characterization have been previously reported.[1]

1.2. IC_{50} data for promysalin and 2

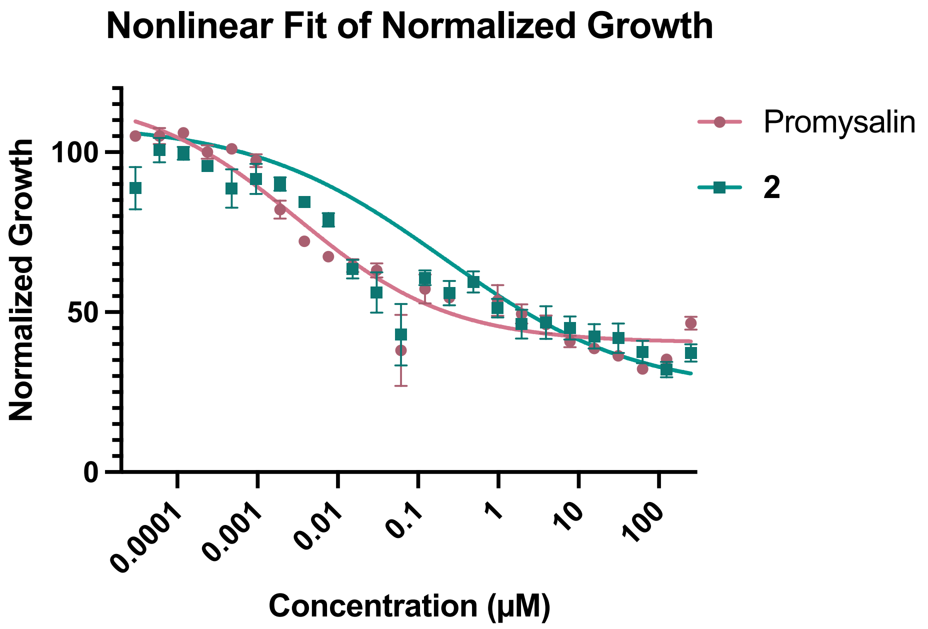

Figure S1: Growth inhibition curve of promysalin and **2** used in this study. Normalized growth and SEM for each data point is shown.

1.3. Equation for calculating FIC_{50} index.

$$\frac{A}{IC_{50_A}} + \frac{B}{IC_{50_B}} = FIC_{50_A} + FIC_{50_B} = FIC_{50}\ Index$$

Equation S1. Fractional inhibitory concentration equation that has been modified for IC_{50} values instead of MIC thus yielding an FIC_{50} index.

2. Chemistry

2.1. General Methods

NMR spectra were obtained using the following spectrometers: Varian INOVA 600 (600/150 MHz), Varian INOVA 500 (500/125 MHz), Bruker 600 (600/125 MHz), or Varian INOVA 400 (400/100 MHz). Chemical shifts are in ppm relative to TMS and use the indicated solvent as

an internal reference. The following abbreviations are used to describe signal multiplicities: s (singlet), d (doublet), t (triplet), q (quartet), m (multiplet), br (broad), dd (doublet of doublets), dt (doublet of triplets), etc. Accurate mass spectra were recorded on a ThermoScientific Exactive Plus Orbitrap MS.

Non-aqueous reactions were performed under an atmosphere of argon, in flame- dried glassware, with HPLC-grade solvents dried by passage through activated alumina. 2,6-lutidine, triethylamine, and diisopropylethylamine were freshly distilled from CaH_2 prior to use. Brine refers to a saturated aqueous solution of sodium chloride, sat. $NaHCO_3$ refers to a saturated aqueous solution of sodium bicarbonate, sat. NH_4Cl refers to a saturated aqueous solution of ammonium chloride, etc. 3Å molecular sieves were activated via heating to 220 °C overnight under vacuum, stored in a 120 °C oven, and flame-dried under vacuum before use. “Column chromatography” refers to purification in a normal-phase gradient on a Biotage® flash chromatography purification system unless noted otherwise. All other chemicals were used as received from Oakwood, TCI America, Sigma-Aldrich, Alfa Aesar, CombiBlocks, or AK Scientific.

2.2. Procedures and Characterization

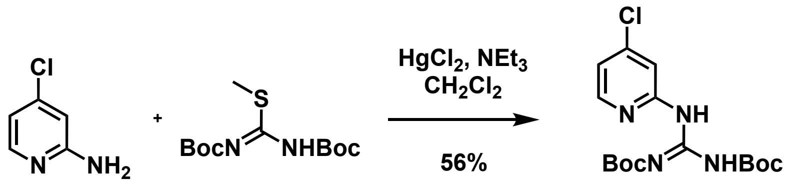

SB002

SB002: To a flask containing commercially available 2-amino-4-chloropyridine (0.100 g, 0.778 mmol) and 1,3-Bis(tert-butoxycarbonyl)-2-methyl-2-thiopseudourea (1.05 equiv, 0.237 g, 0.817 mmol) was added dichloromethane (0.1 M, 7.8 mL) under argon. Cooled to 0˚C and NEt_3 (4 equiv, 0.43 mL) was added followed by $HgCl_2$ (1.1 equiv, 0.232 g, 0.856 mmol). The reaction was stirred at 0˚C for 1 hour and then at room temperature for 2 days. The reaction was diluted with ethyl acetate (10 mL per mmol of pyridine). Solution was filtered through celite and rinsed with ethyl acetate. Combined organics were washed with water then brine. Organics were dried over

Na_2SO_4 and concentrated via rotary evaporation. The crude residue was purified by flash chromatography (gradient of 0 -> 50% diethyl ether in hexanes) to yield pure **SB002** in 56% yield (0.162 g, 0.437 mmol). Characterization data matched those previously reported.[2,3]

^{1}H NMR (500 MHz, CDCl3): δ 11.51 (s, 1H), 10.96 (s, 1H), 8.47 (s, 1H), 8.18 (d, J = 5.4 Hz, 1H), 7.02 (d, J = 5.4 Hz, 1H), 1.53 (s, 18H).

3. Biology

3.1. Raw Synergy Data

3.1.1. Vancomycin

Concentration **2** (µM)

Concentration Vancomycin (µg/mL)	100	50	25	12.5	6.25	3.125	1.563	0.781	0.391	0.195	0.098	0
500	0.093	0.053	0.046	0.046	0.046	0.046	0.046	0.046	0.047	0.059	0.061	0.06
250	0.096	0.053	0.046	0.046	0.046	0.046	0.047	0.083	0.068	0.071	0.071	0.066
125	0.073	0.052	0.046	0.046	0.047	0.053	0.125	0.099	0.097	0.097	0.123	0.17
62.5	0.079	0.056	0.054	0.054	0.066	0.096	0.154	0.166	0.211	0.248	0.281	0.42
31.3	0.104	0.097	0.099	0.102	0.162	0.164	0.228	0.256	0.312	0.406	0.416	0.478
15.6	0.115	0.099	0.122	0.105	0.119	0.146	0.225	0.302	0.346	0.407	0.465	0.518
7.81	0.125	0.102	0.095	0.149	0.11	0.147	0.185	0.29	0.441	0.46	0.516	0.561
0	0.101	0.124	0.118	0.126	0.107	0.149	0.156	0.217	0.318	0.365	0.461	0.539

50% Growth
0.2495

Concentration **2** (µM)

Concentration Vancomycin (µg/mL)	100	50	25	12.5	6.25	3.125	1.563	0.781	0.391	0.195	0.098	0
500	0.05	0.051	0.046	0.046	0.045	0.046	0.046	0.046	0.05	0.06	0.061	0.058
250	0.091	0.05	0.046	0.046	0.046	0.047	0.064	0.085	0.086	0.084	0.067	0.067
125	0.076	0.052	0.047	0.047	0.046	0.071	0.09	0.108	0.102	0.116	0.106	0.177
62.5	0.07	0.052	0.052	0.056	0.064	0.128	0.159	0.173	0.256	0.309	0.311	0.389
31.3	0.1	0.098	0.099	0.101	0.124	0.158	0.207	0.262	0.344	0.461	0.432	0.492
15.6	0.108	0.104	0.096	0.106	0.103	0.131	0.233	0.393	0.43	0.572	0.503	0.544
7.81	0.113	0.109	0.108	0.128	0.138	0.145	0.216	0.236	0.5	0.637	0.583	0.566
0	0.11	0.162	0.157	0.137	0.141	0.192	0.242	0.26	0.484	0.606	0.562	0.633

50% Growth
0.2965

Concentration **2** (µM)

Concentration Vancomycin (µg/mL)	100	50	25	12.5	6.25	3.125	1.563	0.781	0.391	0.195	0.098	0
500	0.104	0.053	0.046	0.046	0.05	0.046	0.046	0.048	0.048	0.06	0.059	0.061
250	0.105	0.054	0.046	0.046	0.046	0.046	0.047	0.077	0.091	0.073	0.096	0.085
125	0.068	0.052	0.047	0.046	0.047	0.049	0.085	0.095	0.11	0.142	0.131	0.185
62.5	0.07	0.055	0.053	0.057	0.087	0.143	0.163	0.199	0.307	0.378	0.345	0.442
31.3	0.109	0.105	0.105	0.124	0.15	0.201	0.215	0.299	0.364	0.48	0.47	0.51
15.6	0.11	0.127	0.102	0.103	0.112	0.186	0.296	0.366	0.403	0.541	0.472	0.552
7.81	0.127	0.144	0.119	0.111	0.108	0.139	0.147	0.205	0.456	0.54	0.494	0.528
0	0.123	0.15	0.145	0.117	0.112	0.158	0.202	0.224	0.271	0.551	0.514	0.596

50% Growth
0.278

3.1.2. Kanamycin

Concentration **2** (µM)

Concentration Kanamycin (µg/mL)	100	50	25	12.5	6.25	3.125	1.563	0.781	0.391	0.195	0.098	0
125	0.087	0.05	0.046	0.045	0.045	0.046	0.046	0.047	0.045	0.045	0.045	0.045
63	0.076	0.051	0.048	0.048	0.047	0.046	0.046	0.046	0.045	0.046	0.045	0.045
31	0.136	0.116	0.111	0.115	0.122	0.117	0.192	0.129	0.111	0.093	0.09	0.046
15.6	0.128	0.13	0.154	0.268	0.252	0.287	0.277	0.259	0.234	0.18	0.164	0.102
7.81	0.131	0.117	0.14	0.221	0.224	0.311	0.295	0.316	0.329	0.376	0.336	0.282
3.91	0.122	0.113	0.111	0.118	0.108	0.188	0.292	0.3	0.354	0.436	0.406	0.503
1.95	0.122	0.113	0.107	0.106	0.103	0.112	0.183	0.158	0.359	0.459	0.481	0.612
0	0.116	0.117	0.136	0.121	0.108	0.106	0.141	0.194	0.212	0.485	0.452	0.612

50% Growth
0.2815

Concentration **2** (µM)

Concentration Kanamycin (µg/mL)	100	50	25	12.5	6.25	3.125	1.563	0.781	0.391	0.195	0.098	0
125	0.078	0.049	0.046	0.045	0.045	0.045	0.045	0.045	0.045	0.045	0.045	0.045
63	0.073	0.048	0.048	0.048	0.046	0.046	0.046	0.045	0.046	0.046	0.045	0.045
31	0.128	0.112	0.122	0.108	0.11	0.123	0.131	0.113	0.117	0.107	0.082	0.06
15.6	0.128	0.131	0.132	0.262	0.266	0.31	0.297	0.289	0.262	0.197	0.166	0.107
7.81	0.125	0.128	0.137	0.282	0.291	0.315	0.387	0.41	0.436	0.397	0.405	0.259
3.91	0.113	0.11	0.122	0.156	0.152	0.287	0.381	0.465	0.456	0.497	0.447	0.517
1.95	0.107	0.103	0.102	0.102	0.105	0.136	0.165	0.479	0.469	0.516	0.529	0.602
0	0.105	0.112	0.136	0.136	0.118	0.146	0.174	0.281	0.449	0.579	0.548	0.614

50% Growth
0.2825

Concentration **2** (µM)

Concentration Kanamycin (µg/mL)	100	50	25	12.5	6.25	3.125	1.563	0.781	0.391	0.195	0.098	0
125	0.092	0.053	0.046	0.046	0.045	0.046	0.045	0.048	0.046	0.047	0.045	0.121
63	0.09	0.05	0.048	0.05	0.049	0.049	0.049	0.047	0.048	0.048	0.047	0.048
31	0.134	0.114	0.116	0.154	0.153	0.195	0.14	0.139	0.129	0.124	0.103	0.066
15.6	0.142	0.138	0.139	0.252	0.271	0.272	0.322	0.306	0.295	0.274	0.193	0.111
7.81	0.148	0.146	0.143	0.253	0.283	0.326	0.36	0.374	0.418	0.42	0.381	0.285
3.91	0.128	0.129	0.121	0.142	0.234	0.278	0.34	0.384	0.424	0.433	0.468	0.567
1.95	0.127	0.137	0.109	0.117	0.11	0.121	0.139	0.168	0.444	0.472	0.572	0.568
0	0.124	0.118	0.125	0.106	0.124	0.126	0.169	0.191	0.219	0.287	0.494	0.628

50% Growth
0.2895

3.1.3. Tobramycin

Concentration **2** (µM); rows: Concentration Tobramycin (µg/mL)

	100	50	25	12.5	6.25	3.125	1.563	0.781	0.391	0.195	0.098	0
2.40	0.069	0.05	0.048	0.048	0.047	0.047	0.047	0.046	0.046	0.047	0.046	0.045
1.20	0.071	0.06	0.053	0.056	0.054	0.051	0.053	0.049	0.047	0.048	0.048	0.046
0.600	0.108	0.122	0.136	0.12	0.125	0.121	0.101	0.096	0.067	0.064	0.135	0.047
0.300	0.168	0.202	0.206	0.223	0.248	0.258	0.263	0.231	0.249	0.274	0.234	0.158
0.150	0.212	0.228	0.228	0.248	0.269	0.289	0.28	0.358	0.385	0.448	0.392	0.335
0.075	0.188	0.208	0.247	0.244	0.241	0.255	0.297	0.382	0.484	0.52	0.486	0.49
0.038	0.196	0.209	0.221	0.226	0.224	0.281	0.348	0.473	0.557	0.562	0.602	0.557
0	0.148	0.149	0.124	0.146	0.162	0.206	0.251	0.463	0.584	0.594	0.601	0.585

50% Growth 0.268

Concentration **2** (µM); rows: Concentration Tobramycin (µg/mL)

	100	50	25	12.5	6.25	3.125	1.563	0.781	0.391	0.195	0.098	0
2.40	0.077	0.051	0.047	0.047	0.046	0.046	0.046	0.046	0.046	0.046	0.046	0.045
1.20	0.075	0.056	0.051	0.051	0.05	0.049	0.048	0.047	0.046	0.047	0.048	0.047
0.600	0.089	0.071	0.116	0.098	0.098	0.101	0.076	0.062	0.059	0.054	0.052	0.047
0.300	0.151	0.17	0.21	0.215	0.226	0.239	0.25	0.226	0.261	0.227	0.2	0.159
0.150	0.125	0.175	0.219	0.233	0.219	0.253	0.301	0.362	0.442	0.378	0.361	0.301
0.075	0.118	0.176	0.224	0.216	0.208	0.269	0.324	0.404	0.492	0.521	0.494	0.482
0.038	0.117	0.191	0.207	0.183	0.198	0.267	0.297	0.426	0.488	0.579	0.576	0.538
0	0.107	0.092	0.107	0.108	0.124	0.146	0.184	0.348	0.442	0.566	0.569	0.599

50% Growth 0.275

Concentration **2** (µM); rows: Concentration Tobramycin (µg/mL)

	100	50	25	12.5	6.25	3.125	1.563	0.781	0.391	0.195	0.098	0
2.40	0.073	0.05	0.047	0.046	0.046	0.046	0.045	0.045	0.046	0.046	0.045	0.045
1.20	0.068	0.056	0.052	0.05	0.05	0.049	0.047	0.047	0.047	0.046	0.047	0.045
0.600	0.088	0.088	0.098	0.1	0.114	0.1	0.085	0.063	0.06	0.059	0.054	0.048
0.300	0.133	0.157	0.189	0.226	0.228	0.218	0.24	0.252	0.232	0.245	0.221	0.127
0.150	0.103	0.18	0.215	0.231	0.233	0.234	0.329	0.343	0.373	0.391	0.371	0.284
0.075	0.103	0.173	0.212	0.227	0.219	0.226	0.328	0.378	0.403	0.461	0.445	0.473
0.038	0.1	0.159	0.165	0.154	0.183	0.239	0.32	0.394	0.445	0.483	0.495	0.538
0	0.105	0.111	0.114	0.123	0.119	0.131	0.176	0.19	0.35	0.419	0.528	0.557

50% Growth 0.254

3.1.4. Gentamicin

Concentration **2** (µM); rows: Concentration Gentamicin (µg/mL)

	100	50	25	12.5	6.25	3.125	1.563	0.781	0.391	0.195	0.098	0
2.40	0.074	0.049	0.047	0.046	0.046	0.046	0.045	0.046	0.046	0.046	0.046	0.045
1.20	0.068	0.054	0.051	0.05	0.05	0.051	0.05	0.074	0.049	0.048	0.047	0.051
0.60	0.102	0.114	0.119	0.127	0.126	0.138	0.112	0.272	0.18	0.173	0.154	0.18
0.30	0.152	0.112	0.145	0.207	0.253	0.234	0.235	0.257	0.256	0.233	0.202	0.201
0.15	0.146	0.104	0.21	0.249	0.259	0.247	0.301	0.365	0.362	0.355	0.344	0.267
0.075	0.145	0.1	0.16	0.135	0.167	0.224	0.301	0.459	0.438	0.483	0.428	0.42
0.038	0.123	0.119	0.093	0.102	0.108	0.121	0.29	0.425	0.443	0.499	0.499	0.492
0	0.124	0.147	0.166	0.156	0.115	0.169	0.241	0.55	0.495	0.53	0.517	0.566

50% Growth 0.2585

Concentration **2** (µM); rows: Concentration Gentamicin (µg/mL)

	100	50	25	12.5	6.25	3.125	1.563	0.781	0.391	0.195	0.098	0
2.40	0.085	0.051	0.047	0.046	0.046	0.046	0.045	0.045	0.045	0.045	0.045	0.05
1.20	0.079	0.053	0.052	0.052	0.051	0.049	0.049	0.048	0.057	0.047	0.047	0.045
0.60	0.087	0.112	0.118	0.115	0.12	0.127	0.13	0.182	0.309	0.159	0.164	0.194
0.30	0.134	0.11	0.15	0.209	0.236	0.261	0.291	0.314	0.277	0.308	0.244	0.22
0.15	0.125	0.112	0.205	0.249	0.271	0.317	0.379	0.435	0.434	0.428	0.386	0.336
0.075	0.125	0.101	0.201	0.2	0.279	0.331	0.394	0.541	0.514	0.559	0.477	0.471
0.038	0.135	0.105	0.099	0.106	0.111	0.189	0.354	0.626	0.613	0.605	0.574	0.574
0	0.124	0.141	0.124	0.103	0.115	0.165	0.251	0.605	0.621	0.623	0.596	0.632

50% Growth 0.2915

Concentration **2** (µM); rows: Concentration Gentamicin (µg/mL)

	100	50	25	12.5	6.25	3.125	1.563	0.781	0.391	0.195	0.098	0
2.40	0.091	0.056	0.048	0.048	0.047	0.047	0.047	0.046	0.046	0.046	0.046	0.046
1.20	0.082	0.064	0.058	0.059	0.06	0.059	0.069	0.063	0.059	0.055	0.052	0.046
0.60	0.131	0.121	0.159	0.17	0.193	0.216	0.202	0.211	0.229	0.214	0.186	0.226
0.30	0.162	0.156	0.258	0.225	0.265	0.326	0.338	0.339	0.333	0.323	0.328	0.247
0.15	0.172	0.196	0.283	0.264	0.293	0.344	0.397	0.443	0.444	0.457	0.417	0.411
0.075	0.16	0.179	0.303	0.291	0.294	0.37	0.43	0.524	0.554	0.541	0.538	0.537
0.038	0.161	0.176	0.188	0.218	0.273	0.368	0.457	0.555	0.598	0.57	0.614	0.634
0	0.139	0.132	0.153	0.157	0.136	0.162	0.396	0.647	0.68	0.647	0.627	0.626

50% Growth 0.2885

3.1.5. Amikacin

Concentration **2** (µM)

Concentration Amikacin (µg/mL)	50	25	12.5	6.25	3.125	1.563	0.781	0.391	0.195	0.098	0.049	0
9.77	0.155	0.154	0.164	0.161	0.164	0.169	0.157	0.142	0.178	0.162	0.141	0.187
4.88	0.183	0.178	0.217	0.225	0.181	0.213	0.222	0.240	0.216	0.174	0.203	0.187
2.44	0.209	0.258	0.315	0.276	0.299	0.261	0.194	0.236	0.224	0.243	0.209	0.225
1.22	0.256	0.289	0.327	0.336	0.451	0.408	0.355	0.363	0.328	0.364	0.334	0.314
0.610	0.252	0.280	0.377	0.390	0.589	0.591	0.605	0.606	0.576	0.533	0.571	0.568
0.305	0.244	0.258	0.303	0.373	0.592	0.668	0.660	0.700	0.661	0.640	0.622	0.638
0.153	0.263	0.293	0.333	0.375	0.629	0.692	0.682	0.694	0.707	0.698	0.717	0.711
0	0.283	0.310	0.358	0.431	0.677	0.739	0.692	0.700	0.728	0.712	0.707	0.695

50% Growth
0.302625

Concentration **2** (µM)

Concentration Amikacin (µg/mL)	50	25	12.5	6.25	3.125	1.563	0.781	0.391	0.195	0.098	0.049	0
9.77	0.144	0.144	0.128	0.140	0.140	0.133	0.144	0.126	0.161	0.136	0.133	0.115
4.88	0.136	0.164	0.147	0.164	0.317	0.183	0.158	0.189	0.157	0.191	0.158	0.171
2.44	0.190	0.231	0.293	0.241	0.263	0.207	0.186	0.221	0.191	0.226	0.319	0.138
1.22	0.214	0.258	0.314	0.307	0.404	0.401	0.364	0.332	0.355	0.359	0.352	0.281
0.610	0.221	0.260	0.302	0.354	0.545	0.616	0.561	0.560	0.620	0.538	0.605	0.576
0.305	0.203	0.224	0.298	0.319	0.619	0.671	0.644	0.644	0.649	0.667	0.645	0.650
0.153	0.226	0.297	0.337	0.332	0.626	0.687	0.692	0.652	0.664	0.714	0.726	0.676
0	0.240	0.293	0.327	0.542	0.640	0.709	0.709	0.658	0.697	0.727	0.690	0.702

50% Growth
0.305975

Concentration **2** (µM)

Concentration Amikacin (µg/mL)	50	25	12.5	6.25	3.125	1.563	0.781	0.391	0.195	0.098	0.049	0
9.77	0.175	0.175	0.170	0.181	0.175	0.181	0.181	0.157	0.192	0.174	0.134	0.182
4.88	0.172	0.179	0.214	0.234	0.197	0.232	0.214	0.242	0.218	0.199	0.205	0.185
2.44	0.143	0.186	0.211	0.172	0.204	0.214	0.168	0.194	0.195	0.204	0.164	0.374
1.22	0.206	0.220	0.203	0.180	0.249	0.223	0.231	0.217	0.189	0.213	0.192	0.175
0.610	0.278	0.309	0.378	0.471	0.364	0.336	0.356	0.304	0.309	0.311	0.331	0.220
0.305	0.283	0.280	0.398	0.400	0.470	0.410	0.424	0.444	0.388	0.426	0.389	0.359
0.153	0.298	0.378	0.426	0.506	0.622	0.686	0.655	0.687	0.657	0.568	0.572	0.563
0	0.287	0.318	0.351	0.418	0.609	0.753	0.749	0.741	0.773	0.774	0.735	0.729

50% Growth
0.3193125

3.1.6. Chloramphenicol

Concentration **2** (µM)

Concentration Chloramphenicol (µg/mL)	50	25	12.5	6.25	3.125	1.563	0.781	0.391	0.195	0.098	0.049	0
39.1	0.265	0.276	0.286	0.332	0.324	0.374	0.383	0.393	0.355	0.353	0.370	0.379
19.5	0.215	0.254	0.282	0.322	0.371	0.447	0.422	0.390	0.390	0.378	0.365	0.357
9.77	0.195	0.231	0.283	0.347	0.359	0.443	0.435	0.435	0.433	0.434	0.430	0.417
4.88	0.210	0.276	0.301	0.328	0.367	0.505	0.491	0.483	0.473	0.484	0.480	0.473
2.44	0.214	0.288	0.326	0.381	0.398	0.595	0.588	0.575	0.614	0.582	0.554	0.558
1.22	0.223	0.252	0.331	0.328	0.453	0.633	0.667	0.666	0.645	0.626	0.627	0.634
0.610	0.238	0.310	0.355	0.441	0.549	0.742	0.728	0.727	0.714	0.722	0.721	0.714
0	0.257	0.315	0.400	0.497	0.584	0.775	0.772	0.720	0.763	0.742	0.738	0.705

50% Growth
0.306675

Concentration **2** (µM)

Concentration Chloramphenicol (µg/mL)	50	25	12.5	6.25	3.125	1.563	0.781	0.391	0.195	0.098	0.049	0
39.1	0.249	0.208	0.241	0.279	0.264	0.274	0.278	0.279	0.281	0.284	0.286	0.291
19.5	0.204	0.225	0.240	0.300	0.345	0.352	0.346	0.381	0.344	0.366	0.349	0.379
9.77	0.202	0.205	0.284	0.295	0.398	0.459	0.383	0.433	0.421	0.466	0.423	0.423
4.88	0.223	0.279	0.270	0.333	0.441	0.505	0.507	0.505	0.494	0.513	0.510	0.502
2.44	0.219	0.291	0.308	0.362	0.507	0.591	0.602	0.588	0.590	0.617	0.632	0.583
1.22	0.244	0.285	0.317	0.359	0.623	0.641	0.684	0.685	0.627	0.675	0.635	0.668
0.610	0.250	0.279	0.352	0.452	0.683	0.701	0.718	0.716	0.708	0.729	0.701	0.710
0	0.276	0.263	0.391	0.446	0.687	0.730	0.739	0.729	0.735	0.737	0.741	0.750

50% Growth
0.3289125

Concentration **2** (µM)

Concentration Chloramphenicol (µg/mL)	50	25	12.5	6.25	3.125	1.563	0.781	0.391	0.195	0.098	0.049	0
39.1	0.282	0.244	0.281	0.307	0.292	0.312	0.311	0.311	0.328	0.322	0.326	0.319
19.5	0.227	0.273	0.295	0.340	0.322	0.357	0.334	0.371	0.350	0.366	0.380	0.410
9.77	0.232	0.239	0.357	0.358	0.445	0.522	0.440	0.480	0.453	0.500	0.452	0.454
4.88	0.237	0.285	0.309	0.368	0.505	0.548	0.534	0.540	0.544	0.556	0.530	0.509
2.44	0.241	0.318	0.347	0.471	0.562	0.694	0.643	0.640	0.614	0.652	0.653	0.586
1.22	0.255	0.312	0.366	0.445	0.664	0.662	0.682	0.689	0.650	0.691	0.683	0.657
0.610	0.282	0.321	0.369	0.520	0.687	0.733	0.723	0.732	0.753	0.752	0.718	0.720
0	0.239	0.272	0.346	0.444	0.706	0.715	0.721	0.713	0.712	0.722	0.710	0.705

50% Growth
0.30635

3.1.7. Erythromycin

Concentration **2** (µM)

Concentration Erythromycin (µg/mL)	50	25	12.5	6.25	3.125	1.563	0.781	0.391	0.195	0.098	0.049	0
156	0.164	0.163	0.179	0.181	0.164	0.195	0.187	0.158	0.196	0.176	0.166	0.188
78.1	0.169	0.184	0.205	0.233	0.207	0.268	0.245	0.274	0.234	0.247	0.254	0.260
39.1	0.177	0.203	0.238	0.231	0.290	0.341	0.286	0.363	0.372	0.389	0.362	0.370
19.5	0.219	0.252	0.260	0.263	0.364	0.433	0.453	0.487	0.454	0.496	0.468	0.440
9.77	0.225	0.257	0.315	0.315	0.395	0.509	0.529	0.572	0.569	0.614	0.573	0.568
4.88	0.232	0.251	0.280	0.338	0.456	0.643	0.612	0.649	0.629	0.617	0.599	0.623
2.44	0.257	0.312	0.342	0.404	0.634	0.703	0.667	0.691	0.711	0.704	0.693	0.675
0	0.295	0.331	0.395	0.486	0.678	0.798	0.752	0.780	0.794	0.795	0.712	0.755

50% Growth
0.3315375

Concentration **2** (µM)

Concentration Erythromycin (µg/mL)	50	25	12.5	6.25	3.125	1.563	0.781	0.391	0.195	0.098	0.049	0
156	0.173	0.188	0.197	0.205	0.190	0.201	0.196	0.172	0.215	0.190	0.174	0.213
78.1	0.174	0.212	0.228	0.245	0.210	0.264	0.272	0.279	0.231	0.237	0.244	0.226
39.1	0.190	0.237	0.276	0.256	0.336	0.370	0.328	0.373	0.350	0.381	0.346	0.365
19.5	0.224	0.267	0.275	0.303	0.397	0.440	0.476	0.465	0.423	0.484	0.457	0.423
9.77	0.232	0.267	0.330	0.348	0.486	0.511	0.532	0.533	0.537	0.550	0.511	0.539
4.88	0.225	0.264	0.291	0.340	0.552	0.607	0.596	0.620	0.598	0.587	0.585	0.600
2.44	0.265	0.298	0.333	0.413	0.660	0.661	0.645	0.629	0.649	0.671	0.647	0.677
0	0.264	0.322	0.382	0.585	0.755	0.785	0.747	0.741	0.757	0.768	0.716	0.748

50% Growth
0.3281375

Concentration **2** (µM)

Concentration Erythromycin (µg/mL)	50	25	12.5	6.25	3.125	1.563	0.781	0.391	0.195	0.098	0.049	0
156	0.236	0.220	0.266	0.254	0.237	0.288	0.285	0.253	0.291	0.236	0.246	0.225
78.1	0.216	0.294	0.270	0.296	0.313	0.337	0.312	0.364	0.378	0.401	0.416	0.313
39.1	0.268	0.258	0.325	0.327	0.384	0.472	0.414	0.459	0.435	0.464	0.383	0.427
19.5	0.265	0.323	0.310	0.403	0.474	0.503	0.508	0.523	0.536	0.568	0.560	0.509
9.77	0.277	0.307	0.367	0.397	0.534	0.607	0.633	0.631	0.610	0.633	0.617	0.558
4.88	0.274	0.277	0.375	0.366	0.583	0.633	0.636	0.691	0.646	0.684	0.631	0.633
2.44	0.282	0.346	0.379	0.433	0.694	0.703	0.707	0.710	0.722	0.693	0.701	0.707
0	0.293	0.320	0.385	0.564	0.726	0.786	0.799	0.760	0.768	0.789	0.745	0.759

50% Growth
0.3335

3.1.8. Tetracycline

Concentration **2** (µM)

Concentration Tetracycline (µg/mL)	50	25	12.5	6.25	3.125	1.563	0.781	0.391	0.195	0.098	0.049	0
31.30	0.186	0.161	0.212	0.214	0.192	0.244	0.239	0.207	0.240	0.214	0.199	0.193
15.65	0.167	0.256	0.242	0.257	0.255	0.292	0.215	0.233	0.392	0.328	0.418	0.213
7.83	0.186	0.195	0.263	0.284	0.279	0.338	0.257	0.293	0.268	0.327	0.267	0.288
3.91	0.219	0.292	0.312	0.379	0.386	0.377	0.352	0.357	0.363	0.399	0.423	0.344
1.96	0.257	0.291	0.348	0.432	0.497	0.498	0.500	0.466	0.480	0.504	0.503	0.406
0.978	0.261	0.261	0.452	0.538	0.634	0.620	0.639	0.638	0.635	0.647	0.620	0.596
0.489	0.254	0.392	0.395	0.649	0.796	0.736	0.720	0.740	0.717	0.702	0.736	0.717
0	0.286	0.322	0.439	0.806	0.783	0.758	0.771	0.740	0.754	0.771	0.754	0.809

50% Growth
0.3595875

Concentration **2** (µM)

Concentration Tetracycline (µg/mL)	50	25	12.5	6.25	3.125	1.563	0.781	0.391	0.195	0.098	0.049	0
31.30	0.206	0.159	0.148	0.163	0.193	0.135	0.147	0.177	0.161	0.138	0.135	0.116
15.65	0.148	0.143	0.182	0.171	0.213	0.179	0.153	0.182	0.143	0.177	0.136	0.165
7.83	0.126	0.175	0.201	0.174	0.225	0.204	0.193	0.214	0.199	0.223	0.219	0.184
3.91	0.177	0.198	0.253	0.252	0.309	0.277	0.289	0.278	0.271	0.285	0.283	0.256
1.96	0.177	0.208	0.294	0.309	0.296	0.320	0.262	0.267	0.263	0.291	0.295	0.292
0.978	0.207	0.217	0.289	0.495	0.593	0.578	0.555	0.559	0.583	0.618	0.555	0.565
0.489	0.216	0.274	0.515	0.647	0.678	0.650	0.678	0.640	0.670	0.675	0.663	0.665
0	0.228	0.275	0.366	0.703	0.767	0.805	0.769	0.703	0.732	0.753	0.752	0.752

50% Growth
0.3308875

Concentration **2** (µM)

Concentration Tetracycline (µg/mL)	50	25	12.5	6.25	3.125	1.563	0.781	0.391	0.195	0.098	0.049	0
31.30	0.131	0.147	0.149	0.156	0.153	0.148	0.164	0.133	0.183	0.148	0.146	0.121
15.65	0.125	0.153	0.154	0.165	0.157	0.161	0.153	0.151	0.151	0.184	0.141	0.169
7.83	0.133	0.152	0.190	0.165	0.206	0.175	0.193	0.194	0.188	0.235	0.338	0.324
3.91	0.196	0.215	0.236	0.268	0.294	0.301	0.308	0.272	0.308	0.297	0.308	0.262
1.96	0.207	0.247	0.267	0.411	0.405	0.422	0.387	0.372	0.411	0.421	0.432	0.373
0.978	0.217	0.230	0.303	0.499	0.580	0.566	0.565	0.579	0.589	0.606	0.561	0.542
0.489	0.236	0.280	0.334	0.674	0.680	0.663	0.673	0.637	0.662	0.665	0.715	0.621
0	0.245	0.301	0.353	0.705	0.731	0.764	0.766	0.716	0.709	0.739	0.735	0.719

50% Growth
0.314475

3.1.9. Ciprofloxacin

Concentration Ciprofloxacin (µg/mL) \ Concentration **2** (µM)	50	25	12.5	6.25	3.125	1.563	0.781	0.391	0.195	0.098	0.049	0
0.610	0.161	0.133	0.141	0.154	0.118	0.133	0.137	0.150	0.129	0.136	0.124	0.121
0.305	0.129	0.133	0.149	0.154	0.177	0.157	0.127	0.190	0.146	0.147	0.141	0.125
0.153	0.129	0.116	0.140	0.151	0.174	0.182	0.163	0.201	0.162	0.212	0.186	0.180
0.076	0.148	0.160	0.231	0.219	0.211	0.229	0.191	0.189	0.171	0.201	0.180	0.141
0.038	0.184	0.232	0.244	0.294	0.438	0.493	0.540	0.491	0.463	0.480	0.488	0.381
0.019	0.198	0.199	0.289	0.327	0.565	0.566	0.566	0.602	0.590	0.567	0.598	0.605
0.010	0.225	0.280	0.286	0.391	0.620	0.688	0.708	0.682	0.644	0.696	0.677	0.666
0	0.249	0.269	0.342	0.379	0.682	0.729	0.715	0.679	0.695	0.677	0.683	0.708

50% Growth
0.3078375

Concentration Ciprofloxacin (µg/mL) \ Concentration **2** (µM)	50	25	12.5	6.25	3.125	1.563	0.781	0.391	0.195	0.098	0.049	0
0.610	0.231	0.185	0.215	0.217	0.212	0.259	0.251	0.221	0.264	0.225	0.207	0.201
0.305	0.214	0.248	0.214	0.262	0.321	0.276	0.257	0.267	0.262	0.295	0.335	0.243
0.153	0.219	0.189	0.248	0.272	0.290	0.321	0.265	0.302	0.259	0.299	0.232	0.271
0.076	0.231	0.270	0.304	0.350	0.369	0.342	0.305	0.342	0.311	0.360	0.354	0.276
0.038	0.262	0.277	0.344	0.430	0.597	0.588	0.605	0.587	0.576	0.619	0.587	0.484
0.019	0.268	0.281	0.377	0.455	0.683	0.660	0.675	0.701	0.675	0.715	0.665	0.635
0.010	0.272	0.348	0.427	0.521	0.766	0.744	0.724	0.766	0.755	0.733	0.789	0.744
0	0.307	0.313	0.421	0.572	0.730	0.784	0.807	0.783	0.764	0.784	0.774	0.748

50% Growth
0.3278875

Concentration Ciprofloxacin (µg/mL) \ Concentration **2** (µM)	50	25	12.5	6.25	3.125	1.563	0.781	0.391	0.195	0.098	0.049	0
0.610	0.242	0.215	0.243	0.244	0.225	0.275	0.273	0.239	0.280	0.253	0.235	0.227
0.305	0.215	0.286	0.257	0.289	0.315	0.304	0.283	0.278	0.298	0.307	0.328	0.247
0.153	0.233	0.228	0.295	0.285	0.300	0.358	0.261	0.285	0.268	0.310	0.262	0.304
0.076	0.243	0.315	0.285	0.304	0.339	0.308	0.284	0.295	0.301	0.341	0.327	0.290
0.038	0.270	0.308	0.404	0.466	0.583	0.626	0.636	0.569	0.574	0.594	0.618	0.514
0.019	0.280	0.302	0.370	0.515	0.654	0.651	0.668	0.691	0.645	0.710	0.665	0.641
0.010	0.290	0.362	0.433	0.607	0.765	0.755	0.736	0.792	0.780	0.725	0.788	0.791
0	0.290	0.305	0.404	0.592	0.744	0.752	0.798	0.717	0.755	0.774	0.747	0.743

50% Growth
0.325575

3.1.10. Levofloxacin

Concentration Levofloxacin (µg/mL) \ Concentration **2** (µM)	100	50	25	12.5	6.25	3.125	1.563	0.781	0.391	0.195	0.098	0
2.40	0.066	0.048	0.048	0.047	0.045	0.046	0.046	0.046	0.045	0.12	0.05	0.046
1.20	0.069	0.055	0.051	0.051	0.048	0.046	0.047	0.048	0.046	0.047	0.05	0.045
0.60	0.085	0.069	0.069	0.073	0.072	0.061	0.085	0.108	0.058	0.092	0.098	0.046
0.30	0.101	0.093	0.106	0.105	0.102	0.104	0.104	0.107	0.117	0.123	0.127	0.1
0.15	0.112	0.111	0.138	0.143	0.135	0.153	0.194	0.218	0.267	0.269	0.278	0.213
0.08	0.124	0.146	0.174	0.162	0.182	0.228	0.288	0.36	0.405	0.448	0.425	0.428
0.04	0.135	0.121	0.127	0.133	0.156	0.215	0.32	0.387	0.514	0.543	0.518	0.502
0	0.134	0.133	0.141	0.137	0.141	0.157	0.162	0.475	0.588	0.553	0.538	0.626

50% Growth
0.2885

Concentration Levofloxacin (µg/mL) \ Concentration **2** (µM)	100	50	25	12.5	6.25	3.125	1.563	0.781	0.391	0.195	0.098	0
2.40	0.068	0.049	0.048	0.047	0.046	0.046	0.046	0.046	0.045	0.046	0.045	0.045
1.20	0.068	0.056	0.054	0.051	0.048	0.046	0.046	0.046	0.046	0.046	0.045	0.045
0.60	0.085	0.07	0.073	0.073	0.07	0.065	0.064	0.057	0.063	0.053	0.066	0.046
0.30	0.108	0.094	0.103	0.103	0.102	0.105	0.106	0.11	0.114	0.121	0.127	0.092
0.15	0.121	0.11	0.131	0.132	0.15	0.167	0.175	0.201	0.233	0.283	0.266	0.231
0.08	0.113	0.139	0.182	0.175	0.19	0.214	0.264	0.3	0.37	0.444	0.431	0.428
0.04	0.116	0.154	0.188	0.183	0.258	0.263	0.302	0.425	0.521	0.556	0.58	0.543
0	0.132	0.118	0.132	0.133	0.119	0.147	0.168	0.509	0.69	0.674	0.644	0.637

50% Growth
0.294

Concentration Levofloxacin (µg/mL) \ Concentration **2** (µM)	100	50	25	12.5	6.25	3.125	1.563	0.781	0.391	0.195	0.098	0
2.40	0.077	0.049	0.048	0.047	0.046	0.046	0.046	0.046	0.045	0.046	0.045	0.045
1.20	0.083	0.055	0.056	0.052	0.049	0.046	0.047	0.046	0.046	0.046	0.047	0.046
0.60	0.09	0.071	0.073	0.074	0.071	0.063	0.061	0.061	0.062	0.048	0.051	0.047
0.30	0.113	0.092	0.101	0.104	0.109	0.113	0.11	0.111	0.116	0.132	0.139	0.105
0.15	0.122	0.105	0.131	0.145	0.16	0.169	0.184	0.236	0.256	0.267	0.265	0.252
0.08	0.12	0.138	0.177	0.182	0.21	0.229	0.281	0.402	0.412	0.438	0.432	0.434
0.04	0.127	0.172	0.193	0.211	0.25	0.26	0.308	0.507	0.552	0.557	0.558	0.567
0	0.115	0.133	0.137	0.142	0.131	0.142	0.192	0.617	0.616	0.618	0.615	0.603

50% Growth
0.277

3.1.11. Trimethoprim

Concentration Trimethoprim (µg/mL) \ Concentration **2** (µM)	25	12.5	6.25	3.125	1.563	0.781	0.391	0.195	0.098	0.049	0.024	0
375	0.075	0.067	0.05	0.049	0.047	0.075	0.085	0.115	0.07	0.047	0.09	0.048
188	0.096	0.096	0.073	0.051	0.054	0.051	0.075	0.053	0.052	0.164	0.05	0.176
94	0.113	0.11	0.107	0.066	0.126	0.239	0.139	0.237	0.196	0.215	0.122	0.106
47	0.127	0.121	0.123	0.214	0.249	0.213	0.234	0.227	0.223	0.16	0.158	0.138
23.4	0.147	0.13	0.143	0.348	0.352	0.379	0.386	0.375	0.419	0.411	0.219	0.196
11.7	0.134	0.147	0.166	0.268	0.444	0.572	0.6	0.616	0.578	0.56	0.497	0.419
5.9	0.126	0.156	0.198	0.224	0.428	0.551	0.585	0.629	0.635	0.672	0.619	0.537
0	0.139	0.151	0.153	0.175	0.295	0.704	0.698	0.672	0.678	0.682	0.645	0.625

50% Growth
0.29

Concentration Trimethoprim (µg/mL) \ Concentration **2** (µM)	25	12.5	6.25	3.125	1.563	0.781	0.391	0.195	0.098	0.049	0.024	0
375	0.077	0.066	0.049	0.05	0.077	0.048	0.048	0.048	0.046	0.115	0.051	0.06
188	0.097	0.1	0.066	0.108	0.1	0.084	0.089	0.167	0.063	0.051	0.166	0.056
94	0.112	0.124	0.099	0.14	0.146	0.157	0.146	0.137	0.069	0.14	0.119	0.136
47	0.129	0.122	0.14	0.246	0.341	0.237	0.202	0.247	0.257	0.186	0.172	0.151
23.4	0.13	0.138	0.163	0.347	0.405	0.391	0.356	0.36	0.383	0.421	0.239	0.17
11.7	0.156	0.161	0.218	0.362	0.575	0.474	0.575	0.56	0.54	0.659	0.543	0.499
5.9	0.134	0.167	0.177	0.176	0.52	0.635	0.625	0.627	0.656	0.647	0.667	0.656
0	0.149	0.149	0.155	0.203	0.466	0.599	0.753	0.728	0.715	0.731	0.727	0.636

50% Growth
0.2955

Concentration Trimethoprim (µg/mL) \ Concentration **2** (µM)	25	12.5	6.25	3.125	1.563	0.781	0.391	0.195	0.098	0.049	0.024	0
375	0.077	0.067	0.049	0.051	0.055	0.049	0.048	0.048	0.107	0.1	0.126	0.049
188	0.106	0.106	0.067	0.054	0.051	0.05	0.164	0.098	0.063	0.053	0.08	0.072
94	0.12	0.127	0.104	0.067	0.149	0.227	0.145	0.096	0.13	0.23	0.113	0.097
47	0.157	0.179	0.168	0.203	0.225	0.224	0.208	0.231	0.231	0.252	0.229	0.201
23.4	0.182	0.216	0.222	0.294	0.321	0.355	0.321	0.325	0.366	0.365	0.351	0.237
11.7	0.199	0.22	0.255	0.343	0.448	0.507	0.551	0.524	0.552	0.57	0.513	0.577
5.9	0.202	0.21	0.229	0.392	0.471	0.663	0.695	0.669	0.706	0.695	0.706	0.706
0	0.183	0.208	0.182	0.321	0.358	0.716	0.7	0.734	0.756	0.726	0.816	0.869

50% Growth
0.412

3.1.12. Polymyxin B

Concentration Polymyxin B (µg/mL) \ Concentration **2** (µM)	25	12.5	6.25	3.125	1.563	0.781	0.391	0.195	0.098	0.049	0.024	0
5	0.044	0.045	0.044	0.043	0.043	0.044	0.096	0.044	0.044	0.054	0.045	0.049
2	0.053	0.046	0.045	0.047	0.044	0.044	0.044	0.044	0.043	0.043	0.044	0.047
1	0.045	0.052	0.045	0.045	0.046	0.075	0.044	0.044	0.044	0.044	0.045	0.043
0.6	0.049	0.058	0.053	0.055	0.135	0.043	0.059	0.044	0.047	0.049	0.044	0.043
0.28	0.119	0.241	0.299	0.334	0.448	0.318	0.275	0.189	0.118	0.055	0.187	0.044
0.14	0.151	0.282	0.324	0.448	0.569	0.48	0.489	0.512	0.521	0.517	0.508	0.559
0.07	0.199	0.298	0.324	0.46	0.612	0.559	0.593	0.612	0.617	0.592	0.618	0.599
0	0.132	0.231	0.274	0.373	0.61	0.594	0.638	0.63	0.593	0.614	0.598	0.579

50% Growth
0.265

Concentration Polymyxin B (µg/mL) \ Concentration **2** (µM)	25	12.5	6.25	3.125	1.563	0.781	0.391	0.195	0.098	0.049	0.024	0
4.50	0.044	0.057	0.043	0.045	0.062	0.048	0.044	0.046	0.045	0.045	0.044	0.043
2.25	0.044	0.049	0.045	0.043	0.044	0.045	0.045	0.044	0.043	0.044	0.051	0.044
1.13	0.05	0.046	0.045	0.046	0.044	0.044	0.046	0.049	0.043	0.044	0.043	0.043
0.56	0.046	0.049	0.045	0.046	0.044	0.043	0.171	0.043	0.044	0.045	0.043	0.043
0.28	0.102	0.231	0.239	0.351	0.379	0.358	0.311	0.327	0.332	0.276	0.299	0.047
0.14	0.118	0.305	0.325	0.405	0.508	0.519	0.564	0.515	0.534	0.561	0.518	0.623
0.07	0.149	0.277	0.314	0.438	0.533	0.594	0.612	0.623	0.615	0.647	0.614	0.638
0.00	0.1	0.13	0.233	0.319	0.506	0.594	0.604	0.586	0.558	0.589	0.628	0.62

50% Growth
0.2855

Concentration Polymyxin B (µg/mL) \ Concentration **2** (µM)	25	12.5	6.25	3.125	1.563	0.781	0.391	0.195	0.098	0.049	0.024	0
5	0.044	0.05	0.044	0.045	0.043	0.044	0.046	0.044	0.049	0.043	0.044	0.043
2	0.045	0.062	0.045	0.044	0.044	0.044	0.045	0.061	0.065	0.043	0.063	0.061
1	0.045	0.047	0.044	0.044	0.044	0.044	0.044	0.043	0.044	0.043	0.044	0.043
0.6	0.047	0.064	0.048	0.045	0.045	0.044	0.044	0.043	0.044	0.044	0.043	0.043
0.28	0.093	0.103	0.268	0.308	0.355	0.263	0.322	0.304	0.312	0.197	0.118	0.043
0.14	0.115	0.201	0.358	0.398	0.486	0.488	0.522	0.491	0.518	0.532	0.517	0.559
0.07	0.106	0.188	0.335	0.48	0.546	0.534	0.565	0.591	0.605	0.606	0.574	0.611
0	0.108	0.119	0.127	0.196	0.442	0.445	0.539	0.554	0.538	0.566	0.546	0.598

50% Growth
0.2745

3.1.13. Colistin

Concentration **2** (µM)

Concentration Colistin (µg/mL)	50	25	12.5	6.25	3.125	1.563	0.781	0.391	0.195	0.098	0.049	0
11.2	0.046	0.047	0.045	0.046	0.045	0.046	0.047	0.046	0.045	0.046	0.047	0.044
5.58	0.047	0.047	0.047	0.046	0.046	0.046	0.048	0.047	0.046	0.047	0.047	0.047
2.79	0.048	0.049	0.048	0.049	0.176	0.05	0.325	0.217	0.323	0.048	0.296	0.054
1.40	0.099	0.098	0.155	0.168	0.42	0.419	0.416	0.404	0.39	0.381	0.366	0.263
0.698	0.186	0.347	0.295	0.374	0.86	0.869	0.888	0.821	0.833	0.832	0.807	0.802
0.349	0.243	0.38	0.372	0.892	0.932	0.948	1.038	0.95	1.002	0.966	0.976	0.928
0.174	0.241	0.323	0.344	0.882	0.984	0.98	1.084	1.065	1.062	1.044	1.052	0.989
0	0.189	0.191	0.206	0.927	1.019	1.009	1.168	1.068	1.032	1.053	1.077	1.042

50% Growth
0.4965

Concentration **2** (µM)

Concentration Colistin (µg/mL)	50	25	12.5	6.25	3.125	1.563	0.781	0.391	0.195	0.098	0.049	0
11.2	0.046	0.047	0.046	0.045	0.045	0.047	0.047	0.047	0.045	0.046	0.047	0.044
5.58	0.046	0.047	0.047	0.047	0.046	0.046	0.047	0.046	0.046	0.046	0.045	0.044
2.79	0.048	0.047	0.048	0.048	0.049	0.048	0.049	0.047	0.047	0.196	0.048	0.054
1.40	0.048	0.049	0.053	0.079	0.169	0.204	0.224	0.072	0.27	0.306	0.266	0.241
0.698	0.143	0.206	0.261	0.317	0.529	0.774	0.829	0.816	0.811	0.809	0.789	0.775
0.349	0.185	0.257	0.315	0.356	0.815	0.876	0.88	0.886	0.911	0.889	0.906	0.848
0.174	0.204	0.2	0.229	0.373	0.869	0.916	0.938	0.953	0.945	0.951	0.974	0.901
0	0.267	0.197	0.241	0.225	0.868	0.893	0.931	0.961	0.938	0.97	0.931	0.919

50% Growth
0.435

Concentration **2** (µM)

Concentration Colistin (µg/mL)	50	25	12.5	6.25	3.125	1.563	0.781	0.391	0.195	0.098	0.049	0
11.2	0.047	0.047	0.045	0.046	0.045	0.046	0.046	0.046	0.044	0.046	0.046	0.044
5.58	0.046	0.047	0.046	0.046	0.046	0.046	0.047	0.047	0.046	0.047	0.049	0.046
2.79	0.058	0.05	0.049	0.048	0.048	0.047	0.054	0.049	0.051	0.05	0.048	0.057
1.40	0.049	0.05	0.051	0.064	0.052	0.198	0.195	0.173	0.175	0.082	0.234	0.489
0.698	0.067	0.258	0.263	0.302	0.382	0.86	0.866	0.9	0.85	0.902	0.915	0.874
0.349	0.231	0.336	0.337	0.363	0.599	1.034	1.001	1.02	0.987	1.028	1.058	0.965
0.174	0.21	0.204	0.245	0.385	0.775	1.029	1.062	1.04	1.007	1.033	1.034	0.971
0	0.193	0.165	0.18	0.226	0.875	1.025	1.041	1.028	1.093	1.071	1.094	0.975

50% Growth
0.463

3.1.14. Piperacillin

Concentration **2** (µM)

Concentration Piperacillin (µg/mL)	25	12.5	6.25	3.125	1.563	0.781	0.391	0.195	0.098	0.049	0.024	0
16	0.102	0.326	0.176	0.165	0.165	0.157	0.151	0.148	0.16	0.146	0.133	0.134
8	0.12	0.388	0.22	0.159	0.159	0.151	0.138	0.137	0.137	0.134	0.133	0.142
4	0.109	0.379	0.23	0.164	0.155	0.14	0.134	0.138	0.139	0.141	0.137	0.138
2	0.131	0.365	0.223	0.182	0.151	0.15	0.287	0.138	0.316	0.147	0.191	0.144
1	0.123	0.353	0.335	0.698	0.659	0.726	0.764	0.787	0.811	0.61	0.869	0.627
0.5	0.275	0.579	0.789	0.714	0.717	0.602	0.741	0.626	0.61	0.714	0.845	0.736
0.25	0.324	0.511	0.807	0.807	0.705	0.704	0.688	0.699	0.687	0.694	0.689	0.607
0	0.161	0.156	0.246	0.611	0.764	0.74	0.783	0.754	0.746	0.757	0.887	0.778

50% Growth
0.3665

Concentration **2** (µM)

Concentration Piperacillin (µg/mL)	25	12.5	6.25	3.125	1.563	0.781	0.391	0.195	0.098	0.049	0.024	0
16	0.177	0.318	0.253	0.185	0.182	0.165	0.163	0.148	0.164	0.153	0.146	0.131
8	0.139	0.264	0.288	0.179	0.171	0.149	0.161	0.154	0.137	0.143	0.132	0.134
4	0.238	0.421	0.273	0.185	0.165	0.147	0.149	0.149	0.14	0.146	0.144	0.136
2	0.133	0.274	0.319	0.199	0.184	0.159	0.154	0.152	0.163	0.148	0.182	0.142
1	0.19	0.309	0.417	0.736	0.756	0.722	0.702	0.72	0.693	0.617	0.588	0.736
0.5	0.405	0.631	0.808	0.748	0.666	0.593	0.62	0.529	0.578	0.597	0.559	0.518
0.25	0.285	0.548	0.813	0.782	0.719	0.677	0.666	0.655	0.65	0.645	0.651	0.593
0	0.153	0.163	0.195	0.627	0.631	0.627	0.638	0.624	0.597	0.625	0.559	0.639

50% Growth
0.297

Concentration **2** (µM)

Concentration Piperacillin (µg/mL)	25	12.5	6.25	3.125	1.563	0.781	0.391	0.195	0.098	0.049	0.024	0
16	0.11	0.261	0.179	0.175	0.168	0.179	0.16	0.147	0.154	0.149	0.129	0.128
8	0.127	0.285	0.185	0.152	0.148	0.135	0.135	0.139	0.144	0.136	0.14	0.127
4	0.108	0.29	0.194	0.157	0.149	0.148	0.142	0.149	0.15	0.151	0.13	0.158
2	0.109	0.287	0.2	0.162	0.156	0.141	0.141	0.144	0.333	0.273	0.23	0.163
1	0.214	0.312	0.355	0.667	0.719	0.655	0.619	0.778	0.691	0.678	0.564	0.674
0.5	0.36	0.51	1.146	0.761	0.682	0.643	0.669	0.541	0.549	0.692	0.662	0.877
0.25	0.328	0.487	0.826	0.754	0.685	0.679	0.664	0.669	0.675	0.66	0.627	0.625
0	0.131	0.139	0.169	0.567	0.697	0.683	0.699	0.7	0.726	0.705	0.69	0.735

50% Growth
0.345

3.1.15. Ceftazidime

Concentration **2** (µM)

Concentration Ceftazidime (µg/mL)	25	12.5	6.25	3.125	1.563	0.781	0.391	0.195	0.098	0.049	0.024	0
16	0.082	0.291	0.144	0.124	0.132	0.129	0.122	0.116	0.124	0.123	0.115	0.111
8	0.087	0.367	0.201	0.144	0.132	0.129	0.119	0.12	0.114	0.124	0.113	0.104
4	0.095	0.359	0.249	0.152	0.144	0.129	0.128	0.125	0.143	0.123	0.111	0.106
2	0.122	0.338	0.274	0.377	0.189	0.191	0.164	0.158	0.187	0.201	0.14	0.123
1	0.255	0.47	0.667	0.81	0.948	0.87	0.845	0.866	0.839	0.849	0.791	0.739
0.5	0.285	0.516	0.808	1.256	0.738	0.698	0.84	0.845	0.807	0.849	0.874	0.704
0.25	0.208	0.23	0.574	0.8	0.768	0.722	0.735	0.745	0.724	0.75	0.8	0.74
0	0.205	0.22	0.217	0.31	0.789	0.733	0.682	0.728	0.75	0.754	0.746	0.751

50% Growth 0.353

Concentration **2** (µM)

Concentration Ceftazidime (µg/mL)	25	12.5	6.25	3.125	1.563	0.781	0.391	0.195	0.098	0.049	0.024	0
16	0.292	0.427	0.215	0.135	0.152	0.146	0.141	0.145	0.132	0.121	0.115	0.128
8	0.134	0.473	0.365	0.164	0.144	0.119	0.127	0.119	0.13	0.124	0.123	0.118
4	0.138	0.364	0.429	0.188	0.157	0.137	0.142	0.131	0.136	0.146	0.129	0.123
2	0.227	0.456	0.422	0.372	0.252	0.187	0.265	0.183	0.144	0.183	0.158	0.148
1	0.229	0.557	0.795	0.921	0.911	0.876	0.768	0.793	0.781	0.768	0.739	0.719
0.5	0.258	0.509	0.781	1.06	0.701	0.747	0.757	0.895	0.862	0.919	0.876	1.085
0.25	0.206	0.251	0.468	0.83	0.75	0.735	0.704	0.698	0.697	0.697	0.695	0.627
0	0.228	0.171	0.193	0.313	0.65	0.625	0.608	0.629	0.595	0.619	0.618	0.624

50% Growth 0.2895

Concentration **2** (µM)

Concentration Ceftazidime (µg/mL)	25	12.5	6.25	3.125	1.563	0.781	0.391	0.195	0.098	0.049	0.024	0
16	0.183	0.299	0.163	0.136	0.153	0.138	0.14	0.134	0.136	0.133	0.13	0.136
8	0.208	0.383	0.217	0.161	0.161	0.146	0.155	0.152	0.142	0.149	0.138	0.126
4	0.124	0.379	0.254	0.157	0.138	0.13	0.135	0.134	0.129	0.131	0.151	0.124
2	0.105	0.356	0.272	0.419	0.246	0.232	0.176	0.174	0.17	0.221	0.165	0.128
1	0.369	0.409	0.725	0.956	0.909	0.858	0.837	0.811	0.807	0.788	0.812	0.738
0.5	0.391	0.526	0.926	1.054	0.697	0.749	0.814	0.846	0.965	1.099	1.224	0.712
0.25	0.217	0.409	0.548	0.757	0.735	0.717	0.696	0.683	0.725	0.708	0.686	0.786
0	0.174	0.148	0.206	0.322	0.717	0.704	0.719	0.701	0.698	0.74	0.727	0.682

50% Growth 0.3185

3.1.16. SB002

Concentration **2** (µM)

Concentration SB002 (µg/mL)	25	12.5	6.25	3.125	1.563	0.781	0.391	0.195	0.098	0.049	0.024	0
22.2	0.561	0.568	0.52	0.503	0.543	0.491	0.493	0.498	0.506	0.487	0.486	0.481
11.1	0.353	0.645	0.677	0.653	0.581	0.59	0.575	0.544	0.573	0.594	0.581	0.637
5.56	0.244	0.4	0.694	0.695	0.631	0.62	0.582	0.577	0.612	0.597	0.658	0.654
2.78	0.228	0.327	0.485	0.636	0.641	0.632	0.595	0.554	0.535	0.578	0.67	0.6
1.39	0.203	0.264	0.303	0.435	0.63	0.643	0.587	0.63	0.599	0.626	0.585	0.662
0.695	0.188	0.271	0.276	0.354	0.487	0.591	0.52	0.643	0.579	0.588	0.613	0.678
0.348	0.194	0.208	0.232	0.324	0.39	0.493	0.504	0.549	0.55	0.563	0.589	0.572
0	0.185	0.201	0.232	0.233	0.324	0.342	0.429	0.586	0.508	0.566	0.713	0.663

50% Growth 0.307

Concentration **2** (µM)

Concentration SB002 (µg/mL)	25	12.5	6.25	3.125	1.563	0.781	0.391	0.195	0.098	0.049	0.024	0
22.2	0.486	0.472	0.496	0.47	0.5	0.478	0.464	0.51	0.461	0.461	0.477	0.481
11.1	0.267	0.606	0.631	0.648	0.625	0.59	0.631	0.564	0.559	0.559	0.583	0.577
5.56	0.234	0.387	0.581	0.619	0.593	0.616	0.597	0.59	0.612	0.63	0.589	0.627
2.78	0.225	0.338	0.585	0.583	0.601	0.6	0.573	0.574	0.57	0.587	0.591	0.607
1.39	0.147	0.244	0.402	0.493	0.609	0.593	0.59	0.631	0.601	0.618	0.622	0.602
0.695	0.152	0.215	0.25	0.351	0.404	0.529	0.559	0.619	0.588	0.61	0.679	0.59
0.348	0.139	0.16	0.201	0.269	0.319	0.405	0.448	0.597	0.603	0.648	0.624	0.597
0	0.134	0.157	0.153	0.265	0.258	0.353	0.418	0.473	0.555	0.652	0.704	0.845

50% Growth 0.398

Concentration **2** (µM)

Concentration SB002 (µg/mL)	25	12.5	6.25	3.125	1.563	0.781	0.391	0.195	0.098	0.049	0.024	0
22.2	0.384	0.452	0.48	0.486	0.47	0.466	0.455	0.545	0.43	0.478	0.443	0.464
11.1	0.274	0.461	0.508	0.556	0.512	0.522	0.487	0.567	0.546	0.544	0.52	0.511
5.56	0.271	0.325	0.63	0.557	0.618	0.55	0.576	0.554	0.593	0.583	0.559	0.579
2.78	0.234	0.308	0.495	0.586	0.617	0.586	0.581	0.575	0.532	0.527	0.517	0.542
1.39	0.203	0.225	0.34	0.482	0.568	0.611	0.585	0.57	0.602	0.58	0.552	0.655
0.695	0.202	0.174	0.199	0.259	0.458	0.599	0.544	0.54	0.573	0.494	0.55	0.571
0.348	0.196	0.198	0.21	0.173	0.297	0.376	0.485	0.462	0.512	0.554	0.516	0.509
0	0.17	0.142	0.131	0.165	0.198	0.326	0.499	0.435	0.65	0.467	0.677	0.573

50% Growth 0.262

3.1.17. PAβN

Concentration **2** (μM)

Concentration PAβN (μg/mL)	25	12.5	6.25	3.125	1.563	0.781	0.391	0.195	0.098	0.049	0.024	0
40	0.104	0.11	0.107	0.116	0.13	0.147	0.168	0.157	0.182	0.195	0.178	0.281
20	0.138	0.19	0.196	0.204	0.248	0.3	0.301	0.343	0.343	0.37	0.391	0.508
10	0.185	0.225	0.283	0.275	0.398	0.464	0.477	0.509	0.529	0.556	0.519	0.571
5.0	0.203	0.233	0.309	0.383	0.475	0.643	0.605	0.662	0.649	0.646	0.631	0.618
2.50	0.256	0.243	0.323	0.387	0.543	0.731	0.688	0.722	0.738	0.747	0.718	0.74
1.25	0.254	0.268	0.34	0.424	0.589	0.72	0.762	0.755	0.735	0.786	0.734	0.794
0.63	0.207	0.283	0.282	0.429	0.565	0.763	0.765	0.744	0.764	0.774	0.764	0.797
0	0.232	0.269	0.297	0.409	0.735	0.735	0.757	0.701	0.702	0.677	0.686	0.789

50% Growth
0.370

Concentration **2** (μM)

Concentration PAβN (μg/mL)	25	12.5	6.25	3.125	1.563	0.781	0.391	0.195	0.098	0.049	0.024	0
40	0.106	0.111	0.111	0.112	0.117	0.15	0.14	0.178	0.168	0.209	0.204	0.291
20	0.143	0.188	0.21	0.231	0.253	0.284	0.303	0.307	0.33	0.382	0.352	0.52
10	0.189	0.263	0.218	0.285	0.398	0.48	0.528	0.521	0.526	0.588	0.577	0.565
5.0	0.222	0.324	0.274	0.316	0.482	0.651	0.683	0.689	0.691	0.69	0.669	0.635
2.50	0.276	0.274	0.261	0.323	0.537	0.713	0.746	0.747	0.724	0.756	0.733	0.796
1.25	0.22	0.245	0.309	0.382	0.595	0.728	0.759	0.718	0.741	0.728	0.755	0.784
0.63	0.207	0.255	0.31	0.335	0.636	0.723	0.71	0.697	0.726	0.728	0.744	0.791
0	0.191	0.253	0.257	0.384	0.618	0.706	0.678	0.663	0.622	0.659	0.732	0.742

50% Growth
0.347

Concentration **2** (μM)

Concentration PAβN (μg/mL)	25	12.5	6.25	3.125	1.563	0.781	0.391	0.195	0.098	0.049	0.024	0
40	0.096	0.108	0.109	0.113	0.118	0.085	0.127	0.132	0.161	0.174	0.176	0.303
20	0.123	0.151	0.226	0.271	0.258	0.306	0.321	0.339	0.326	0.357	0.317	0.535
10	0.139	0.208	0.239	0.288	0.341	0.462	0.492	0.495	0.528	0.555	0.513	0.531
5.0	0.192	0.258	0.293	0.376	0.506	0.633	0.647	0.643	0.672	0.661	0.684	0.647
2.50	0.216	0.255	0.254	0.4	0.512	0.676	0.662	0.628	0.734	0.708	0.725	0.73
1.25	0.225	0.238	0.249	0.349	0.502	0.681	0.784	0.735	0.753	0.742	0.703	0.749
0.63	0.185	0.206	0.265	0.353	0.473	0.676	0.623	0.747	0.695	0.745	0.74	0.764
0	0.177	0.203	0.235	0.328	0.617	0.614	0.664	0.749	0.626	0.708	0.741	0.808

50% Growth
0.380

4. References

(1) Steele, A. D.; Keohane, C. E.; Knouse, K. W.; Rossiter, S. E.; Williams, S. J.; Wuest, W. M. Diverted Total Synthesis of Promysalin Analogs Demonstrates That an Iron-Binding Motif Is Responsible for Its Narrow-Spectrum Antibacterial Activity. *J Am Chem Soc* **2016**, *138* (18), 5833–5836.

(2) McVey, A. C.; Bartlett, S.; Kajbaf, M.; Pellacani, A.; Gatta, V.; Tammela, P.; Spring, D. R.; Welch, M. 2-Aminopyridine Analogs Inhibit Both Enzymes of the Glyoxylate Shunt in Pseudomonas Aeruginosa. *Int J Mol Sci* **2020**, *21* (7).

(3) Dickinson, R. P.; Barber, C. G. 2-Pyridinylguanidine Urokinase Inhibitors. US 6583162 B1, 2003.
